# Integrated Isogenic Human Induced Pluripotent Stem Cell–Based Liver and Heart Microphysiological Systems Predict Unsafe Drug–Drug Interaction

**DOI:** 10.3389/fphar.2021.667010

**Published:** 2021-05-07

**Authors:** Felipe T. Lee-Montiel, Alexander Laemmle, Verena Charwat, Laure Dumont, Caleb S. Lee, Nathaniel Huebsch, Hideaki Okochi, Matthew J. Hancock, Brian Siemons, Steven C. Boggess, Ishan Goswami, Evan W. Miller, Holger Willenbring, Kevin E. Healy

**Affiliations:** ^1^ Departments of Bioengineering, and Materials Science & Engineering, University of California Berkeley, Berkeley, CA, United States; ^2^ Department of Surgery, Division of Transplant Surgery, Liver Center and Eli and Edythe Broad Center of Regeneration Medicine and Stem Cell Research, University of California San Francisco, San Francisco, CA, United States; ^3^ Institute of Clinical Chemistry and Department of Pediatrics, Inselspital, University Hospital Bern, Bern, Switzerland; ^4^ Department of Bioengineering and Therapeutic Sciences, Schools of Pharmacy and Medicine, University of California San Francisco, San Francisco, CA, United States; ^5^ Veryst Engineering, LLC, Needham, MA, United States; ^6^ Department of Chemistry, University of California Berkeley, Berkeley, CA, United States; ^7^ Departments of Chemistry and Molecular & Cell Biology, and Helen Wills Neuroscience Institute, University of California Berkeley, Berkeley, CA, United States

**Keywords:** drug–drug interaction, microphysiological systems, liver and heart integration, ketoconazole, cisapride, hiPSC-derived cells

## Abstract

Three-dimensional (3D) microphysiological systems (MPSs) mimicking human organ function *in vitro* are an emerging alternative to conventional monolayer cell culture and animal models for drug development. Human induced pluripotent stem cells (hiPSCs) have the potential to capture the diversity of human genetics and provide an unlimited supply of cells. Combining hiPSCs with microfluidics technology in MPSs offers new perspectives for drug development. Here, the integration of a newly developed liver MPS with a cardiac MPS—both created with the same hiPSC line—to study drug–drug interaction (DDI) is reported. As a prominent example of clinically relevant DDI, the interaction of the arrhythmogenic gastroprokinetic cisapride with the fungicide ketoconazole was investigated. As seen in patients, metabolic conversion of cisapride to non-arrhythmogenic norcisapride in the liver MPS by the cytochrome P450 enzyme CYP3A4 was inhibited by ketoconazole, leading to arrhythmia in the cardiac MPS. These results establish integration of hiPSC-based liver and cardiac MPSs to facilitate screening for DDI, and thus drug efficacy and toxicity, isogenic in the same genetic background.

## Introduction

The drug development pipeline generally starts with a broad collection of candidate compounds that are narrowed down by traditional *in vitro* drug screening tools including biochemical analysis and cell-based assays, for example, using immortalized cell lines, computational modeling, and then testing in animal models. The successful compounds are then tested in human preclinical and clinical trials. The costs for pharmaceutical companies to develop a new drug from initial compound screening to market are estimated at more than 2.5 billion USD (Herper, 2013; DiMasi et al., 2016) and can take ∼10–15 years (Mathur et al., 2013; Mathur et al., 2015). One of the more costly and inefficient steps in this process is the clinical trial phase (ASPE, 2018). Too often compounds pass the go/no-go risk decisions based on animal model data, but fail later in clinical trials due to species-specific differences in physiology. The worst-case scenario occurs when drugs make it through the full screening pipeline and clinical trials to market, only to be recalled due to unforeseen side effects in some patients, a prominent example being the arrhythmogenic effect of the gastroprokinetic cisapride (Okada et al., 2015). In addition to the inability of animal models to accurately mimic human physiology, such failures are due to lack of diversity in the human genetic backgrounds tested in single cell lines, and lack of diversity in phase I and II clinical trials.

Drug-induced liver injury (DILI) and cardiotoxicity are the leading causes of preclinical and clinical withdrawals of marketed pharmaceuticals due to safety issues (Kaplowitz, 2005; Guo et al., 2011; Craveiro et al., 2020), and manifest as serious complications, including hepatitis and liver cell death, potentially leading to liver failure, and cardiac arrhythmia (Abboud and Kaplowitz, 2007; Ewer and Ewer, 2015). The human liver is especially significant for toxicity as it is the first stop for most compounds after they enter the body for initial drug metabolism before subsequent uptake by other organs (The International Transporter Consortium, 2010). Animal models perform variably in representing human diseases and predicting toxicity to humans due to inter-species differences in drug absorption, distribution, metabolism, excretion, and/or toxicity (ADME/Tox) (Jianling and Suzanne, 2009; Scott et al., 2013; Proctor et al., 2017). Accordingly, the risk of human DILI is not well predicted by animal models. Additionally, animal studies can only partially recapitulate patient-specific factors such as genetics, age, environment, and concomitant diseases (Olson et al., 2000).

The limitation of animal models to predict DILI has led to the development of *in vitro* liver models; however, current versions have limitations, most notably due to the complexity of the liver, which performs more than 500 functions, including metabolic, synthetic, immunologic, and detoxification processes (Taub, 2004). The most commonly used models of liver toxicity involve two-dimensional (2D) monolayer cultures of primary human hepatocytes (pHeps). They currently represent the standard for drug screening, toxicology studies, cell-based therapies, and *in vitro* disease modeling. Indeed, they recapitulate most liver functions and represent diverse human genetic disease backgrounds that are not captured by single human cancer cell lines. pHeps also bypass ethical concerns regarding refined and reduced use of animal experiments. However, there are still substantial problems using pHeps. One of the limitations is the difficulty to perform long-term cultures (≥7 days) due to the propensity of pHeps to dedifferentiate *in vitro* within a few days, causing them to lose hepatocyte-specific functions (Hewitt et al., 2007; Rowe et al., 2013) and no longer accurately represent the biology of the liver. Co-cultures of pHeps with endothelial cells and/or fibroblasts, as well as recent advances in long-term cultures of pHeps, might overcome these limitations (Nahmias et al., 2006; Xiang et al., 2019), but not the shortage of donor livers (Fraczek et al., 2013; Griffith et al., 2014), resulting in high cost and considerable genetic and functional variation of commercial pHeps (Bhushan et al., 2016). These variations alone make interlaboratory comparisons nearly impossible. Taken together, conventional 2D monolayer cultures using pHeps are often too simplified, ultimately failing to adequately predict DILI, putting human lives at risk and wasting valuable resources for pharmaceutical companies during clinical drug development.

Human induced pluripotent stem cell (hiPSC)-derived hepatocytes (hiPSC-Heps) have the potential to overcome the limitations of pHeps and human cell lines. hiPSCs are pluripotent cells generated by reprogramming of mature somatic cells, for example, adult human fibroblasts, by overexpression of the Yamanaka factors (Takahashi and Yamanaka, 2006; Takahashi et al., 2007). hiPSCs can provide a virtually inexhaustible source of cells from different genotypes, including healthy or disease-specific cells (Si-Tayeb et al., 2010; Hannan et al., 2013). By providing a renewable source of cells that maintain the genotype of the donor, hiPSCs reduce cell source variability and facilitate an unlimited number of trials of patient-specific drug screening at the early stage of the development pipeline (Liu et al., 2010; Choi et al., 2011; Liu et al., 2011; Mi et al., 2013). However, like other protocols for directed differentiation of hiPSCs, current differentiation protocols for hiPSC-Heps produce immature cells that lack certain functions of pHeps (Shan et al., 2013; Avior et al., 2015).

Although pHeps and hiPSC-Heps are widely used in 2D monolayer culture for initial assessment of drug metabolism, these monocultures do not provide 3D organization, non-parenchymal cells, structural cell–cell interactions, nor their associated paracrine interactions (Bale et al., 2014). Advances in 2D co-culture (e.g., of hiPSC-Heps and fibroblasts) exploiting micropatterned architecture have shown promising results predicting DILI (Berger et al., 2014). Compared to 2D, bioinspired 3D *in vitro* systems provide more structural organization, which is beneficial to maintaining cell differentiation and maturity (Gieseck et al., 2014; Ardalani et al., 2019). For example, 3D culture can reverse or prevent dedifferentiation of pHeps (Dunn et al., 1991; Dash et al., 2009) and promote metabolic enzyme functionality comparable to *in vivo* levels for adult rat hepatocytes (Tuschl et al., 2009; Ebrahimkhani et al., 2014; Proctor et al., 2017). Thus, the combination of hiPSCs and 3D culture offers great potential to improve the efficiency and efficacy of the drug development pipeline by allowing to study hepatocytes in a more biologically relevant setting (Dalgetty et al., 2009; Fabre et al., 2014; Kyungsuk et al., 2014; Moradi et al., 2020). However, only few published studies used hiPSC-Heps to build 3D liver MPSs (Bale et al., 2014; Mun et al., 2019; Bircsak et al., 2021).

Another challenge in the study of DDI is inter-organ cross talk. Specifically, liver metabolism is a key modulator of drug toxicity in various other organs. Several recent studies have tackled the challenge of linking liver models to other organ and tissue models (Lee and Sung, 2018; Boeri et al., 2019; Baert et al., 2020; Pires de Mello et al., 2020). Since cardiotoxicity is the most common cause for safety-related market withdrawal, in this study we focused on heart–liver cross talk as one clinically relevant organ pair. Liver and heart models have been linked by a few research groups in recent years (Zhang et al., 2017; Oleaga et al., 2018; McAleer et al., 2019; Pires de Mello et al., 2020); however, in these combination platforms the individual tissue models were either 2D monolayer cultures (Zhang et al., 2017; McAleer et al., 2019; Pires de Mello et al., 2020), very simple organoids (Zhang et al., 2017), or genetically distinct (Zhang et al., 2017; Oleaga et al., 2018; McAleer et al., 2019; Pires de Mello et al., 2020). We and others have demonstrated that 3D liver and cardiac MPS are functionally superior to conventional 2D monolayer cultures (Bale et al., 2014; Mathur et al., 2015; Prodanov et al., 2016; Schepers et al., 2016; Du et al., 2017; Lee-Montiel et al., 2017; Huebsch et al., 2018). To date, an integrated liver–heart MPS generated with cells derived from a single donor has not been reported.

In our study, we present the first use of a 3D hiPSC-based *in vitro* liver model on a microfabricated platform, that is, MPS (also known as “organ-on-a-chip” or “tissue chip”), functionally coupled with a 3D hiPSC-based cardiac MPS platform (Mathur et al., 2015; Huebsch et al., 2018; Tveito et al., 2018). Moreover, the use of isogenic hiPSCs allowed us to produce different 3D tissues—liver and heart—from the same donor and thus with identical genetic background. In addition, we modified our recently reported protocol (Zhu et al., 2014) to produce hiPSC-Heps suitable for studies of drug metabolism, including sufficient cytochrome P450 (CYP) 3A4 activity and long-term function in the MPS. As an example of clinically relevant DDI, we focused on cisapride, a gastroprokinetic intended to treat the symptoms of heartburn in adults (Wiseman and Faulds, 1994). This drug causes prolongation of the QT interval, ventricular arrhythmias, and torsade de pointes (Wysowski and Bacsanyi, 1996) in patients taking medications that interfere with cisapride metabolism or prolong the QT interval. In 2000, cisapride was removed from the US market as a result of 80 deaths and 341 incidents of arrhythmias, including 107 episodes of torsade de pointes, reported to the FDA from 1993 to 1999 (Wysowski et al., 2001; Shah, 2002). To functionally connect the liver and cardiac MPSs, we developed a common medium for survival and function of each tissue. By correctly predicting CYP3A4-dependent DDI between cisapride and the antifungal ketoconazole, we show that MPS technologies are effective for predicting drug efficacy and toxicity across multiple organs, and have potential as next-generation high-content drug development tools.

## Materials and Methods

### Cell Sources

The hiPSC line WTC was generated by reprogramming of skin fibroblasts derived from a healthy 30-year-old Japanese adult male with no known family history of heart disease, generously provided by Prof. Bruce Conklin (Gladstone Institutes) and available from the Coriell Repository (#GM25256). WTC was used for the generation of hiPSC-Heps. hiPSC-derived cardiomyocytes were generated from WTC edited to harbor a single copy of CAG-driven GCaMP6f knocked into the first exon of the AAVS1 “safe harbor” locus (Huebsch et al., 2015). For experiments with pHeps, commercially available cryopreserved cells from ThermoFisher Scientific (lot#Hu8138) were used.

### Differentiation of Human Induced Pluripotent Stem Cell-Heps

Differentiation of hiPSCs (day 0) into hiPSC-Heps (days 23–25) was performed according to an optimized protocol. The cells were cultured on Cultrex Basement Membrane Extract (BME) (Trevigen) diluted 40 times in Knockout DMEM (ThermoFisher Scientific) for the entire hepatocyte differentiation process. At day 0, hiPSCs were split and kept in mTESR (Stem Cell Technologies) supplemented with 10 µM Rock inhibitor (Tocris Bioscience) to form a confluent layer of hiPSCs on day 1 when the hepatocyte differentiation was initiated. From days 1 to 7, RPMI 1640 medium (ThermoFisher Scientific) was used and supplemented with standard antibiotics and antimycotics and recombinant human Activin A (PeproTech EC) at 100 ng/ml. Between days 2 and 7, Gem21 NeuroPlex without Insulin (Gemini BioProducts), nonessential amino acids (ThermoFisher Scientific), and sodium butyrate (Sigma Aldrich) at 500 µM was added. KnockOut Serum Replacement (ThermoFisher Scientific) was added to the differentiation medium from days 1 to 3 in decreasing amounts of 2, 1, and 0.2%, respectively. The small molecules CHIR 99021 (Tocris Bioscience) at 3 µM and PI-103 (Selleckchem) at 50 nM were added at day 1 and from days 1 to 3, respectively. From day 8, the cells were cultured in IMDM (ThermoFischer Scientific) supplemented with standard antibiotics and antimycotics, Gem21 NeuroPlex without Insulin (Gemini BioProducts); 100 nM insulin, 100 nM dexamethasone, and 400 µM monothioglycerol (all Sigma Aldrich); nonessential amino acids; and human FGF2 at 10 ng/ml and human BMP4 at 20 ng/ml (both Peprotech) until day 18. At day 13, HGF (Peprotech) at 20 ng/ml was added until the end of the hepatocyte differentiation. From day 18 onward, the cells were cultured in Hepatocyte Basal Medium (Lonza CC-3199) supplemented with all the components of Hepatocyte Culture Medium SingleQuots except EGF (Lonza CC-4182 containing gentamicin-amphotericin, transferrin, insulin, hydrocortisone, and fatty acid–free bovine serum albumin). This medium is referred to as hepatocyte culture medium (HCM).

### Immunofluorescence in Conventional Cell Culture and Liver Microphysiological System

In conventional cell culture, hiPSCs and hiPSC-Heps were fixed for 20 min at room temperature (RT) in 4% paraformaldehyde (PFA) and washed three times with phosphate-buffered saline (PBS). The cells were blocked for 1 h with 3% BSA and permeabilized with 0.3% Triton X-100 (Sigma). The cells were labeled with rabbit anti-OCT3/4 antibody (sc-9081; SantaCruz Biotechnology) diluted 1:500, goat anti-HNF4 antibody (sc-6557; SantaCruz Biotechnology) diluted 1:500, goat antihuman albumin antibody (A80–129A; Bethyl Laboratories) diluted 1:500, mouse anti–alpha-fetoprotein (MA1-19342; Invitrogen) diluted 1:500, and DAPI (D1306; Invitrogen) for nuclear staining. The secondary antibodies were Alexa Fluor 488/555 donkey-anti-rabbit or anti-goat or anti-mouse IgG diluted 1:1,000 (Invitrogen). The cells were imaged using a Nikon Eclipse TE300 with a Lumencore Spectra X light engine. The liver MPS was washed with PBS and fixed at RT for 30 min with 4% PFA using a 3D collagen gel-staining protocol and washed three times with PBS. After permeabilization in 1% Triton X-100 (Sigma) for 30 min, the cells were incubated for ∼24 h at 4°C with the same primary antibodies as above but at different concentrations: Both goat antihuman albumin and mouse anti–alpha-fetoprotein were diluted 1:100. Secondary antibodies with fluorescent probes Alexa 488 (A-11055; Invitrogen) and R-PE 565 (P21129; Invitrogen) were incubated at 4°C for 6 h and washed three times for 5 min with cold PBS. The liver MPS was imaged by confocal microscopy. The confocal images were collected using a Carl Zeiss LSM 710 confocal microscope equipped with a plan-apochromat 10 Å∼/0.45 objective.

### Flow Cytometry

At day 23 of differentiation, hiPSC-Heps were digested with 1 mg/ml collagenase for 20 min to singularize cells. The cells were then fixed with 4% PFA for 20 min at RT and labeled with the following antibodies: Antihuman albumin (A80–129A; Bethyl Laboratories) detected with a secondary antibody conjugated with Alexa Flour-488 (Life Technologies) and anti-ASGR1 (8D7 clone conjugated with PE; BD Biosciences). Albumin and ASGR1-positive cell populations were determined by flow cytometry analysis with an Attune NxT Acoustic Focusing Cytometer (Thermo Fisher Scientific). FlowJo software was used to analyze the data.

### Drug Transport Study

Cells were washed with carbogenated Williams’ E (Gibco; A1217601) (adjusted pH 7.4 at 37°C) for 15 min at 37°C before transport or metabolism studies. The drug uptake transport study was initiated by adding each transporter substrate with or without its inhibitor in Williams’ E medium, and incubation at 37°C for 2 min in a shaker-incubator. The reaction was terminated by removing the dose solution followed by washing the cells three times with ice-cold PBS. The following substrates and inhibitors were used: 10 µM acyclovir (including 0.1 µCi [^3^H]-acyclovir, OAT substrate) and 1 mM probenecid (OAT inhibitor) for the OAT transport study, 1 mM metformin (including 1 µCi [^14^C]-metformin, OCT substrate) and 1 µM decynium-22 (OCT inhibitor) for the OCT transport study, and 10 µM rosuvastatin (including 0.25 µCi [^3^H]-rosuvastatin, OATP substrate) and 50 µM rifamycin-SV (OATP inhibitor) for the OATP transport study. Cells were harvested for further drug analysis. The drug efflux transport study was initiated by adding each transport substrate with or without its inhibitor in Williams’ E medium and incubation at 37°C for 15 min in a shaker-incubator. The reaction was terminated by removing the dose solution followed by washing cells three times with ice-cold PBS. The following substrates and inhibitors were used: 10 µM digoxin (including 0.25 µCi [^3^H]-digoxin, P-gp substrate) and 10 µM GG918 (P-gp inhibitor) for the P-gp transport study and 10 µM of mitoxantrone (including 1 µCi [^3^H]-mitoxantrone, BCRP substrate) and 5 µM GG918 (BCRP inhibitor) for the BCRP transport study. Cells were harvested for further drug analysis (see “*Drug Metabolism Measurements*” section).

### Cytochrome P450 Luciferase Activity Assay

CYP activity was assessed using the luciferin-based P450-Glo assays (Promega). Cells were washed with basal HCM for 15 min prior to incubation with CYP substrates at the following concentrations: 100 μM for 1A2, 100 μM for 2C9, 10 μM for 2C19, 30 μM for 2D6, 3 μM for 3A4, and 150 μM for 3A7. Cells were incubated with substrates for 3.5 h except for the 3A4 assay that was incubated for 70 min. At the end of the incubation period, 50 µL supernatant was transferred into a 96-well plate in technical triplicates, and 50 µL of CYP-specific detection reagent was added to each well. The plate was covered in foil and mixed gently for 20 min, and absorbance was measured at 450 nm on a plate reader (SpectraMax i3, Molecular Devices). Samples included a negative control consisting of HCM only (no cells) that was used for background correction.

### Drug Metabolism Enzymatic Assays

Each enzyme substrate was dissolved in Williams’ E medium and incubated at 37°C in a shaker-incubator. For metabolism studies of UGTs and SULTs, the cells were incubated with 10 µM 1-naphthol or 10 µM nitrophenol (Sigma-Aldrich), respectively, for 15 min at 37°C. Then the dosing solution was collected; the cells were washed three times with ice-cold PBS and harvested for further drug analysis (see “*Drug Metabolism Measurements*” section).

### Quantitative Polymerase Chain Reaction

RNA was extracted from hiPSC-Heps and pHeps by the Trizol/chloroform method. Each sample was suspended in 500 µL of Trizol to which 100 µL (1 V:5 V) of chloroform was added. After 15 s of vortexing and 5 min of incubation at RT, the samples were centrifuged for 5 min at 12,000 rpm. The upper phase containing RNA was transferred into a new Eppendorf tube. Isopropanol (1 V:1 V) was added to each tube and slowly inverted five times. The samples were centrifuged for 10 min at 12,000 rpm at 4°C. The RNA pellet was kept on ice until the end of the procedure. The pellet was washed twice in 750 µL of 70% ethanol and air-dried on ice. The pellet was resuspended in an appropriate volume of nuclease-free water and the RNA concentration was determined on a Nanodrop (ThermoFisher Scientific). The reverse transcription was performed with 1 μg of RNA template using cDNA Supermix (Quanta Biosciences) and nuclease-free water in a final volume of 20 μl. qPCR was performed on 100 ng of cDNA using SYBR Green Supermix (Affymetrix) and specific primers on an Applied Biosciences ViiA7 Real-Time PCR System (Invitrogen).

### Computational Modeling

Laminar fluid flow and dilute species transport in the liver MPS was simulated using COMSOL Multiphysics® 5.3a (COMSOL AB). Laminar fluid flow was governed by the Navier–Stokes equations and dilute species (oxygen (O_2_) and a generic small molecule) transport by the advection–diffusion equation, both solved in COMSOL® subject to the following boundary conditions: no-slip on all media channel walls and membrane surfaces; laminar inflow rate specified at the media channel inlet; laminar outflow conditions at the outlet; and dilute species concentration specified at the inlet. O_2_ diffusion through the 3.5-mm-thick PDMS slab was also simulated in COMSOL®, subjected to ambient O_2_ concentration at the top of the slab and O_2_ partial pressure continuity at the media channel and cell chamber walls. In our simulations, fluid (media with supplements) with a density of 1,000 kg/m^3^ and a dynamic viscosity of 0.78 mPa·s at 37°C (Bacabac et al., 2005; Mathur et al., 2015) was used. Flow across the 15-μm-thick porous membrane was neglected since low membrane porosity (5.6%), small membrane pore size (mean pore diameter 3 μm), and cultured cells on the cell chamber side of the membrane resulted in flow resistances through the membrane that were orders of magnitude higher than through the media channel (height 100 μm) (Loskill et al., 2017). Moreover, the cultured cells within the cell chamber increased flow resistance there. Thus, it was assumed that there was no media flow into or within the cell chamber so that dilute species transport within the cell chamber was by diffusion only (Loskill et al., 2017). Small flows within cell culture chambers in membrane bilayer microfluidic devices have been considered elsewhere (Inamdar and Borenstein, 2011; Inamdar et al., 2011).

Three sets of COMSOL® simulations were conducted. In the first set, the transport of a generic small molecule within culture media in the liver MPS was modeled at an inlet flow rate of 20 μl/h, an inlet concentration of 1 mol/m^3^, zero initial concentration in the device, a diffusion coefficient of 1.0 × 10^−9^ m^2^/s in culture media, and no cell uptake and no flux through walls. The second and third sets of simulations involved O_2_ transport, diffusion, and uptake. The media channel inlet and initial media channel and cell chamber O_2_ concentrations were 0.173 mol/m^3^, which is the saturation level in culture media at 37°C in equilibrium with ambient incubator air at 37°C, 1 atmosphere (atm) pressure, with 18.7% O_2_ (21% O_2_ in dry air reduced by 5% CO_2_ and 6% water vapor). On the PDMS surfaces exposed to ambient incubator air, the O_2_ concentration in the PDMS was 1.11 mM, the saturation level. The diffusion coefficients of O_2_ in PDMS and culture media were 3.25 × 10^−9^ m^2^/s and 3.0 × 10^−9^ m^2^/s, respectively (Buchwald, 2009; Markov et al., 2014; Mathur et al., 2015). Cell O_2_ consumption was modeled by the Michaelis–Menten kinetics with rate = −VO_2max_ • *ρ*_cell_ • c/(K_m_ • S_cell_ + *c*), where c was the local O_2_ concentration, K_m_ = 5.6 mmHg was the Michaelis–Menten constant (Allen and Bhatia, 2003; Kim et al., 2013), and S_cell_ = 1.049 mM/(atm O_2_) was the solubility of O_2_ in cells (Chakraborty et al., 2007; Kim et al., 2013). VO_2max_ = 1.04 × 10^−16^ mol/s/cell was measured for hiPSC-Heps using the Seahorse XF24 cellular respirometer (Agilent) (see the Oxygen Consumption Rate section for details). A cell density in the cell chamber of *ρ*_cell_ = 6.44 × 10^13^ cells/m^3^ based on ∼ 10,000 (9,812 ± 838) cells was assumed per liver chip.

### Liver Microphysiological System Fabrication and Design Dimensions

The liver MPS was designed to mimic a ∼100 parallel circuit of liver sinusoids (∼6–15 μm in width) (Braet and Wisse, 2002; Vollmar and Menger, 2009). The design consisted of a rectangular chamber divided by a PET membrane to mimic the fenestrations present in the sinusoidal endothelial cells in the human liver (Horn et al., 1987). The liver MPS was composed of two chambers with the following dimensions: 5,560 μm (length) × 560 μm (width) × 100 μm (height) for the cell chamber side, and in the media channel the volume was 0.32 μL. The total volume of the chip was empirically measured to account for the cell inlet and the media outlet having a total volume of 2 μL. Computational modeling (see the previous section) was used to verify the chip dimensions and fluidic design was adequate for the transport of O_2_ and drugs.

The dual chamber microfluidic device was fabricated using replica molding from photolithographically defined SU-8 masters on silicon wafers. Briefly, 30 g (top layer) and 6 g (bottom layer) of PDMS was poured into two silicon wafer masters and baked at 80°C for 8 h, after which O_2_ plasma was used to bond PDMS devices to the PET membrane with 3 μm pore size (Sabeu GmbH) and the cover glass. To adhere to the PET membranes, they were treated with bis [3-(trimethoxysilyl)propyl]amine silane and then rendered hydrophilic (protocol modified from Sip and Folch (2014) and Loskill et al. (2017)). This treatment also allows for PET to remain bonded even in the presence of salt-containing media. The PET membranes were prepared by first being cut into slightly larger rectangle shapes than those of the individual cell or media layer patterns and then cleaned in isopropyl alcohol for 10 min. The membranes were then suspended in a plasma chamber that was filled with 20% O_2_ and exposed to plasma at 60 W for 60 s. The power source used was a PE-1000 AC Plasma power source. The membranes were then incubated in a solution of 97% isopropyl alcohol, 2% bis(3-(trimethoxysilil)propyl)amine, and 1% H_2_O at 80°C for 20 min. Following the incubation, the membranes were rinsed with isopropyl alcohol and dried at 80°C for 30 min. Following drying (*curing*), the membranes were individually placed in 2 ml of 70% ethanol in type I ASTM laboratory reagent grade H_2_O for 30 min to render the membrane hydrophilic.

### Liver Microphysiological System Loading With Human Induced Pluripotent Stem Cell-Heps

Prior to cell loading, the MPS was sterilized by UV light for 30 min, coated with 100 μg/ml human fibronectin (Corning, United States) and 100 μg/ml rat tail collagen, type I (Corning, United States) in PBS, and the interior of the device was dried under vacuum for 30 s. hiPSC-Heps were trypsinized for 8–10 min and quenched using 80% DMEM/F12 (Thermofisher, Carlsbad, CA) with 20% FBS, pelleted at 50 g for 5 min, and then resuspended at 11 × 10^6^ hepatocytes/mL in HCM supplemented with 10% fetal bovine serum (Cellgro) and 10 µM Rock inhibitor. hiPSC-Heps were then pipetted into the cell inlet port of the device and centrifuged in a bucket at 300 × g for 3 min for optimal cell loading. The seeded devices were incubated overnight at 37°C to enhance the formation of a 3D organization. Plating media was removed from the device and HCM was added and incubated for 24 h at 37°C to allow stabilization of the model before initiating a 10 μL/h flow perfusion using a syringe pump (New Era NE-1800).

To quantify the number of total cells in the liver MPS, the confocal images of cell nuclei stained with DAPI in the cell chamber were imaged by z-stack confocal microscopy (Zeiss LSM 710). Then, the Cell Counter plugin of Fiji ImageJ was used to manually count the cells as the automated counter was not able to distinguish cells accurately in 3D. This process was repeated in triplicates and a best estimate of the average cell number (9,812 ± 838 cells) in the liver MPS was used to normalize albumin and urea secretion per cell.

### Live/Dead Assays

To assess viability of hiPSC-Heps in the liver MPS, the LIVE/DEAD Cell Imaging Kit from Molecular Probes (R37601) was used. Viable cells were stained with green-fluorescent calcein AM (488 nm) and dead cells with red ethidium homodimer-1 (570 nm). The liver MPS was rinsed with PBS and 60 μL of dye were added via the media channel and incubated for 20 min. Images were collected using an inverted Nikon TE300 microscope with a Lumencore Spectra X light engine. Images were processed with Fiji ImageJ. Counts of live or dead cells were obtained using a segmentation analysis coupled to a constant area exclusion filter. For long-term culture, the liver MPS was incubated at 37°C and fed by a New Era NE-1800 syringe pump with continuous media flow (10 μL/h). For visual characterization, the cells were imaged using a brightfield phase contrast daily without detachment from the syringe pump using a Nikon Eclipse TE300 microscope.

### Efflux Media Collection and Biochemical Measurements in Liver Microphysiological System

Perfusion of the liver MPS was initiated 2 days after loading to allow recovery and spreading of the hiPSC-Heps. Media was collected every two days and analyzed for albumin and urea secretion. Albumin was measured using an enzyme-linked immunosorbent assay (Bethyl Laboratories). Urea nitrogen was measured using a colorimetric assay (urea nitrogen test, Stanbio Laboratory) modified to be performed in a 384-well microtiter plate, including increase in the incubation time of reactants from 60 to 90 min at 60°C prior to reading. All the biochemical assays were performed in 10 µL of media and measured on a SpectraMax i3 plate reader (Molecular Devices). All the sample results were calculated by interpolation of sample raw values from standard curves performed in parallel. The same assays were repeated for conventional cell cultures of hiPSC-Heps.

### Drug Metabolism of Human Induced Pluripotent Stem Cell-Heps in Conventional Cell Culture and Liver Microphysiological System

For drug metabolism study in conventional cell culture, hiPSC-Heps were incubated with 1 or 10 µM of cisapride (Sigma) in Williams’ E + Cocktail B (see below) with or without 10 µM ketoconazole (Sigma) for the indicated time-points (from 30 min to 24 h) at 37°C in an incubator. Then the dosing solution was collected, the cells were washed three times with ice-cold PBS, and harvested for further drug analysis by mass spectrometry (see “*Drug Metabolism Measurements*” section). To determine CYP3A4 activity and its inhibition by ketoconazole in the liver MPS after day 25, a luminescence assay (P450GLo, Promega) was used. Briefly, the liver MPS was incubated with luciferin-PFBE for 24 h, after which the supernatant was transferred to a 96-well plate and the luciferin detection reagent was added to initiate a luminescent reaction. The plate was then incubated for 20 min at RT to complete the reaction. Luminescence levels were measured on a SpectraMax i3 plate reader. CYP3A4-mediated metabolism of cisapride was performed in Williams’ E + Cocktail B. Media was supplemented with 100 nM and 1,000 nM cisapride and perfused through the liver MPS on differentiation day 26. These concentrations were subsequently corrected for the absorption into PDMS (see “*Drug Absorption to Polydimethylsiloxane*” section). Efflux media was collected over a 24-h period and stored at −80°C for further analysis by mass spectrometry (see “*Drug Metabolism Measurements*” section).

### Drug Metabolism Measurements

The cisapride drug and norcisapride metabolite were extracted from efflux media and analyzed using a Shimadzu UFLC system (Carlsbad) coupled with a Sciex API5000 triple quadrupole mass-spectrometer (Foster City) using positive ionization mode. Cisapride and norcisapride were further separated by a gradient mode of 10 mM ammonium acetate–acetonitrile as a mobile phase using an Acclaim Trinity P1 reverse phase column (2.1 × 50 mm, 3 μm; Thermo Fisher Scientific). Mass ion transitions (Q1/Q3) of cisapride and norcisapride were m/z 468.3/186.0 and m/z 314.2/184.0, respectively. 1-naphthol, 1-naphthol-s-glucuronide, *p*-nitrophenol, and *p*-nitrophenol sulfate were separated by a gradient elution with 0.1% formic acid in H2O and 0.1% formic acid in acetonitrile as mobile phases using a XTerra MS C18 reverse phase column (4.6 × 50 mm, 3 μm; Waters). Mass ion transitions of 1-naphthol, 1-naphthol-s-glucuronide, *p*-nitrophenol, and *p*-nitrophenol sulfate were m/z 143.3/115.3, m/z 318.9/143.3, m/z 138.2/168.1, and m/z 217.9/138.2, respectively. The standard curve range of each drug and metabolite was 0.5–2,000 nM. Precision (defined by the coefficient of variation) and accuracy (defined by relative error) of LC-MS/MS analyses were both <15% for all drugs and metabolites.

### EC_50_ Studies of Cisapride on the Cardiac Microphysiological System

Culture medium for the cardiac MPS was RPMI 1640 medium (11,875–093; Gibco) supplemented with B-27 (17,504–044; Gibco). The cardiac MPS was fabricated as described in previous studies (see Supplementary Material) (Mathur et al., 2015; Huebsch et al., 2018). Detailed characterization of the cardiac MPS including gene profiling, flow cytometry, immunofluorescence, and computational analysis of ion channels has been performed (Mathur et al., 2015; Huebsch et al., 2018; Tveito et al., 2018; Jæger et al., 2019). For all pharmacology, the cardiac MPS was first equilibrated to phenol red–free Williams’ E media + Cocktail B (see below) containing vehicle control (DMSO or water, to a final concentration of 0.1% v/v). On the day of the experiment, the freshly measured cisapride drug was dissolved into DMSO. After the initial baseline recording in vehicle control condition, media was exchanged for the lowest cisapride drug dose, and the cardiac MPS was incubated for 30 min at 37°C. Media changes during the drug testing were either performed manually (Huebsch et al., 2018) or using an automated Fluigent pump system (Fluigent, N. Chelmsford, United States). A volume flow of 30 μL/min was applied for 5 min to change to a new drug dose followed by 15 μL/min continuous perfusion. Spontaneous beating activity was recorded via brightfield microscopy. Optical measurement of action potential in the cardiac MPS was performed using the voltage-sensitive dye Berkeley Red Sensor of Transmembrane potential (BeRST-1; Supplementary Figure S1A) (Huang et al., 2015). BeRST-1 is a far-red to near-infrared dye that changes fluorescence intensity in response to membrane voltage changes through a photo-induced electron transfer mechanism. BeRST-1 dye was synthesized and its purity verified as previously described (Huang et al., 2015). For action potential recording, cardiac MPS was first labeled overnight with 2.5 µM BeRST-1. For the cardiac MPS metrics analysis, the beat rate–corrected action potential durations at 30, 80, and 90% repolarization (cAPD_30_, cAPD_80_, and cAPD_90_; Supplementary Figure S1B) were used.

### Cisapride and Ketoconazole Drug–Drug Interaction Testing in Liver and Cardiac Microphysiological Systems

We used “functional coupling” for the DDI studies, where the media was first exposed to the liver MPS, collected, and then exposed to the cardiac MPS. Functional coupling of the liver and cardiac MPS started with testing of the basal pHep maintenance Williams’ E medium in the cardiac MPS to determine its effect on electrophysiology and toxicity in hiPSC-derived cardiomyocytes prior to DDI studies. Williams’ E medium was supplemented with Gibco Cell Maintenance Supplement Pack B (Cocktail B; Thermofisher Scientific), which contains bovine serum albumin (5.35 μg/ml) as a carrier protein and other supplements such as ITS that support pHep culture. Dexamethasone was not included to exclude likely confounding effects on hiPSC-derived cardiomyocytes.

For the DDI experiment, first the liver MPS was exposed to cisapride (50–100 nM) in Williams’ E basal media + Cocktail B with or without ketoconazole (10 μM) for 8 h at 37°C. The efflux was collected from the media port after passing through the liver MPS and stored at −20°C. Next, the cardiac MPS was equilibrated with phenol red–free Williams’ E media + Cocktail B containing vehicle control (DMSO to a final concentration of 0.1% v/v). After the initial APD baseline recording while exposed to the vehicle control, the cardiac MPS was exposed to the thawed efflux media for 30 min at 37°C, followed by APD measurements as described below.

### Cardiac Microphysiological System Imaging and Analysis

All the images were collected using a Nikon Eclipse TE 300 microscope. The brightfield videos were analyzed for beating physiology using an updated version of our open-source motion-tracking software (Huebsch et al., 2015). The software can be downloaded at https://sites.wustl.edu/huebschlab/resources/. Microscopy files were directly read into the MATLAB-based graphical user interface (GUI), and the contractile motion was analyzed via an exhaustive-search block-matching optical flow algorithm that compared the position of 8 × 8 pixel macroblocks at frame i to their position at frame i+5 (corresponding to the motion in 50 msec). Motion vectors were used to calculate beat rate. BeRST-1 data were quantified using in-house MATLAB code that was developed based on previous work by Laughner et al. (2012) and Boggess et al. (2019). BeRST-1 videos were analyzed for beat rate and 80 and 90% action potential duration (APD_80_, APD_90_), using custom MATLAB and *Python* scripts.

### Characterization of Drug Absorption into Polydimethylsiloxane

While PDMS is widely used to build microfluidic devices, due to its biocompatibility and convenience for microfabrication, the material has the disadvantage that it can absorb small molecules with low LogP values (van Meer et al., 2017; Halldorsson et al., 2015). Thus, absorption of cisapride to PDMS was characterized to correct the dosages used in the experiments. The dose escalation studies were first repeated in the liver MPS, without cells, to model the drug analysis. Using LC-MS/MS, ion abundances of the drug in stock samples and in efflux media were measured to calculate the percent remaining. One device was used per escalation study of 10, 50, 100, 500, and 1,000 nM, and the average percent remaining were 35.52, 30.54, 30.95, 34.55, and 39.79 nM, respectively (Supplementary Figure S2). For the drug response studies, the cisapride concentration was corrected for PDMS drug absorption (60–70%) (Shirure and George, 2017; van Meer et al., 2017).

Details for the drug absorption measurements follow. Assembled liver and cardiac MPS chips were placed on a temperature-controlled glass base (Thermo Plate; Tokai Hit) to maintain the temperature of the chips at approximately 37°C. The drug cisapride was prepared to the concentrations of 5, 10, 50, and 100 nM in Williams’ E medium supplemented with Cocktail B. The MPSs were loaded with 100 *μ*L of drug solution via a pipette tip at the media inlet port and an empty pipette tip placed at the media outlet. Flow at the media channel was induced due to the difference in hydrostatic pressure head at the inlet and the outlet. For a given concentration of drug, the system was given 30 min after loading before the pipette tip at the inlet was taken out and the solution in the device and at the outlet tip was collected. Experiments were started with the lowest concentration (i.e., 5 nM) and dose concentration was increased for subsequent experiments (i.e., dose escalation). Technical replicates for each drug concentration are indicative of solutions collected from different devices. Drug solutions collected from devices were diluted by half using a 10 nM solution of propranolol used as an internal standard for LC-MS/MS. Standards of cisapride drug with propranolol as internal control were also prepared. Samples were analyzed using an LC system (1200 series; Agilent) connected in line with an LTQ-Orbitrap-XL MS equipped with an electrospray ionization source and operated in the positive ion mode (ThermoFisher Scientific). The LC was equipped with a reversed-phase analytical column (length: 150 mm, inner diameter: 1.0 mm, particle size: 5 μm; Viva C18; Restek). Acetonitrile, formic acid (Optima LC-MS grade, 99.5+%; Fisher), and water purified to a resistivity of 18.2 MΩ cm (at 25°C) using a Milli-Q Gradient ultrapure water purification system (Millipore) were used to prepare mobile phase solvents. Data acquisition and processing were performed using Xcalibur software (version 2.0.7, ThermoFisher Scientific). Processed data were used to quantify the amount of drug lost due to absorption in the PDMS-based MPSs via comparison with the drug standards.

### Statistical Analysis

Differences in drug and control population measurements reported as significant have a *p* < 0.05, as determined by the application of a *t*-test with the assumption of equal variance. Direct comparisons were made by nonpaired Student’s *t* test and for multiple comparisons ANOVA was used. All curve fitting was done using GraphPad Prism 6.

## Results

### Directed Differentiation of Human Induced Pluripotent Stem Cell-Heps

**FIGURE 1:**
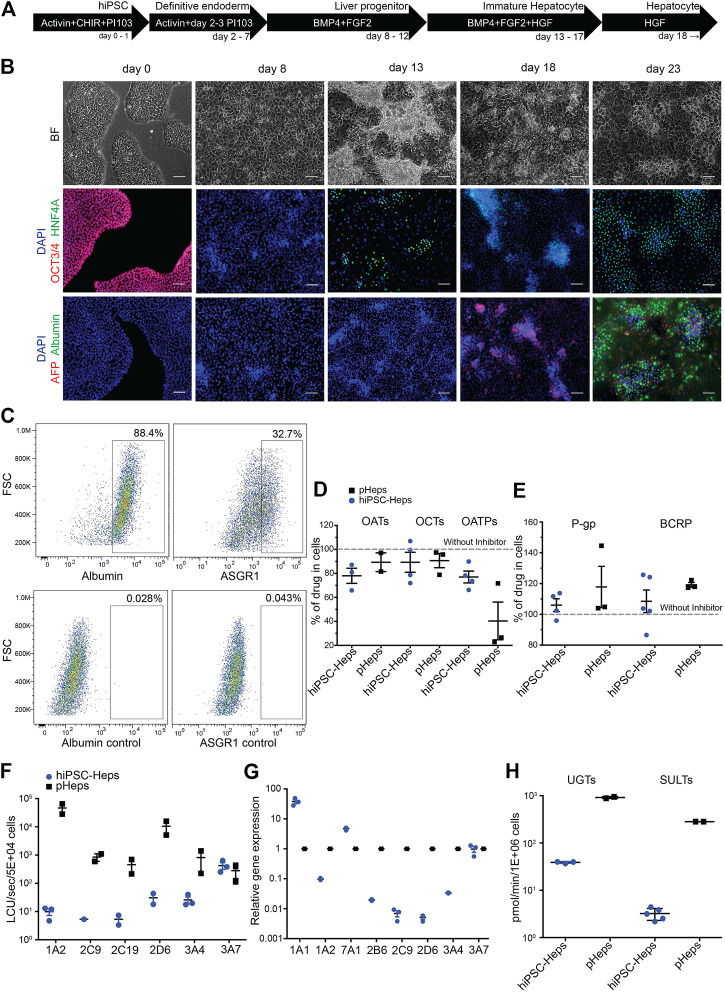
Differentiation and characterization of hiPSC-Heps. **(A)** Schematic of the hiPSC-Hep differentiation protocol. The arrows show cells at different stages progressing from hiPSCs (day 0) to mature hepatocytes (day 23). Growth factors and small molecules are listed inside the arrows. **(B)** Brightfield and immunofluorescent images matching the differentiation stages in **(A)**. Nuclei were stained with DAPI. Scale bars, 100 µm. **(C)** Flow cytometry shows percentage of hiPSC-Heps expressing albumin and ASGR1 at day 23. **(D)** Activity analysis of uptake transporters by inhibition with specific inhibitors. Results obtained without inhibitors were set to 100% to display transporter activity as decrease of drug concentration in the cells. Data are presented as the mean ± standard deviation from three to four (hiPSC-Heps) or two to three (pHeps) independent experiments. **(E)** Activity analysis of efflux transporters by inhibition with specific inhibitors. Results obtained without inhibitors were set to 100% to display transporter activity as increase of drug concentration in the cells. Data are presented as the mean ± standard deviation from four to five (hiPSC-Heps) and three (pHeps) independent experiments. **(F)** Activity analysis of CYPs. Data are presented as the mean ± standard error of the mean from three (hiPSC-Heps) or two (pHeps) independent experiments. **(G)** Quantitative reverse transcription PCR analysis of CYP genes. **(H)** Activity analysis of conjugating enzymes. Data are presented as the mean ± standard deviation from two (UGTs, SULTs; pHeps), three (UGTs; hiPSC-Heps), or five (SULTs; hiPSC-Heps) independent experiments.

Directed differentiation of hiPSCs into hiPSC-Heps was performed in 2D cell culture using an optimized protocol (Figure 1A; see Material and Methods for details). Brightfield microscopy showed characteristic morphological changes occurring during the differentiation process (Figure 1B) from hiPSCs (day 0) to definitive endoderm (day 8), liver progenitors (day 13), immature (day 18), and mature hepatocytes (day 23). Immunofluorescence showed lack of the pluripotency marker OCT3/4 past the hiPSC stage (Figure 1B); hepatic nuclear factor 4 alpha (HNF4A) started to be expressed at day 13 when cells committed to hepatic fate; alpha-fetoprotein (AFP) stained strongly positive in immature hepatocytes at day 18; and albumin (ALB) was only detectable at the final stage of differentiation (day 23). At this stage, 88.4% of cells expressed albumin and 32.7% were positive for asialoglycoprotein receptor 1 (ASGR1), a marker of mature hepatocytes, in flow cytometry (Figure 1C and Supplementary Figure S3).

### Characterization of Drug Metabolism in Human Induced Pluripotent Stem Cell-Heps

A prerequisite for drug metabolism studies is sufficient cellular uptake of the drug as well as efflux of the metabolites. Therefore, activities of three uptake (Figure 1D) and two efflux (Figure 1E) transporters were measured by high-performance liquid chromatography coupled with tandem mass spectrometry (LC-MS/MS) using drug/inhibitor combinations. For the uptake transporter studies, hiPSC-Heps were exposed to a specific drug internalized by the transporter, with or without a specific inhibitor of the transporter—activity of organic anion transporters (OATs) was tested with acyclovir/probenecid, organic cation transporters (OCTs) with metformin/decynium-22, and organic anion transporting polypeptides (OATPs) with rosuvastatin/rifamycin-SV. Metabolism-qualified pHeps were used as controls. All the three phase 0 uptake transporters were active in hiPSC-Heps and pHeps, including responsiveness to specific inhibitors (Figure 1D). Next, the activities of two phase III efflux transporters were analyzed using substrate/inhibitor challenge. The P-glycoprotein (P-gp) was tested with the substrate digoxin and the breast cancer resistance protein (BCRP) with mitoxantrone. Both efflux transporters were active in hiPSC-Heps and pHeps, including responsiveness to the P-gp/BCRP inhibitor GG918 (Figure 1E). To further assess whether our hiPSC-Heps allowed studies of phase I drug metabolism, we measured the activity of key hepatic CYP enzymes (Figure 1F). Based on a luminescence assay, CYP3A4 was one of the most active drug-metabolizing CYPs in hiPSC-Heps and its activity was in the range of 3–5% of pHeps (Figure 1F). Activities of other CYPs were 2–3 orders of magnitude lower in hiPSC-Heps than in pHeps (Figure 1F), which was confirmed by gene expression analysis (Figure 1G) and indicated some lack of maturation of our hiPSC-Heps compared to pHeps. However, relatively high CYP3A4 activity suggested maturation beyond the fetal hepatocyte stage since CYP3A4 is not expressed in fetuses and neonates (Tréluyer et al., 2001). Finally, the activities of the phase II conjugating enzymes UDP-glucuronosyltransferases (UGTs) and sulfotransferases (SULTs) were in the range of 1–10% of pHeps, as shown by LC-MS/MS using the substrate/metabolite combination 1-naphtol/naphtol-s-glucuronide and nitrophenol/nitrophenol-sulfate, respectively (Figure 1H). In summary, our optimized protocol produced hiPSC-Heps capable of all phases of drug metabolism, although some transporter and enzyme activities were underdeveloped. This finding accords with findings made with other protocols for hiPSC-Hep differentiation and underscores the importance of in-depth characterization of these cells to ascertain suitability for the intended experiments.

### Development of the Liver Microphysiological System

**FIGURE 2:**
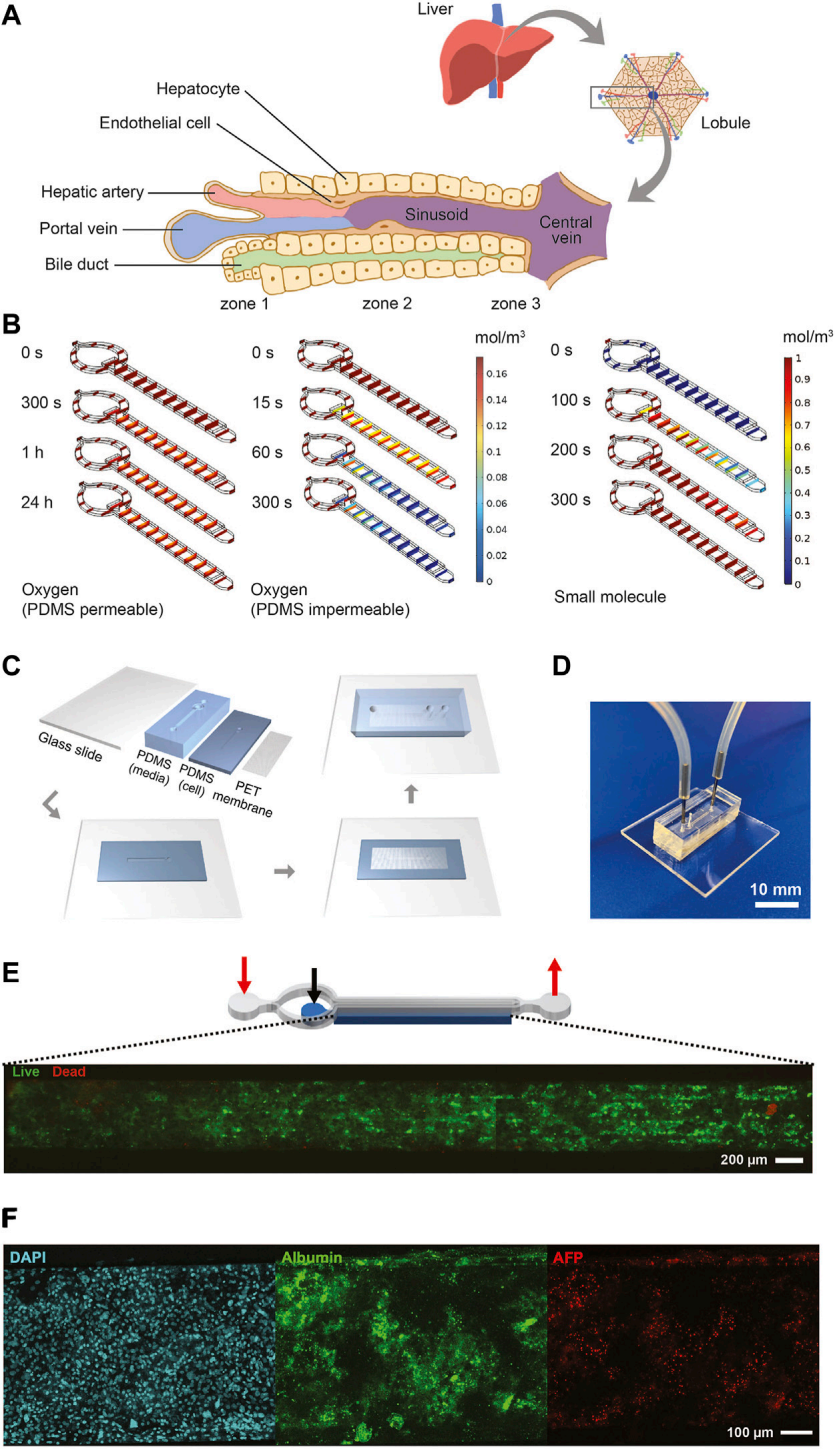
Design, characterization, fabrication, and testing of liver MPS. **(A)** Illustration showing the liver acinus as the basic building block of the liver inspiring the liver MPS design. **(B)** COMSOL® simulation results. Small concentration gradients with physiologically relevant oxygen levels are observed when cells in the cell chamber consume oxygen and oxygen diffuses from the ambient through the PDMS roof and walls **(left)**. When oxygen is not diffused through the PDMS cells become hypoxic after 300 s **(center)**. The hiPSC-Heps OCR used in the simulations was determined from Seahorse measurements. The oxygen concentration jumps between media channel and cell chamber are due to diffusion across the porous membrane. A dilute solution containing a small molecule entering the media channel at 20 μL/h diffuses across the porous membrane into the cell chamber and reaches a uniform concentration within the liver MPS within 300 s **(right)**. Simulation assumes impermeable walls and no cell consumption. **(C)** Preparation and microfabrication steps of the liver MPS. **(D)** Photograph of a ready-to-use liver MPS. The catheter couplers link the tubes and microfluidic channels for perfusion. The short plug in the middle keeps the loaded cells in the chamber. **(E)** Schematic of the liver MPS that shows the localization of the cell chamber; the red arrows indicate the inlet and the outlet of the media channels, whereas the black arrow marks the inlet of the cell chamber. High cell viability in the tissue chamber after loading is demonstrated by fluorescence imaging of acetoxymethyl calcein (calcein-AM; live cells labeled green) and ethidium homodimer (dead cells labeled red). **(F)** Immunofluorescent images of hiPSC-Heps seven days after loading into the liver MPS.

The liver MPS was designed to mimic a single acinus, the smallest functional unit of the liver, which comprises three different zones, exposing hepatocytes to different concentrations of nutrients and oxygen (Figure 2A). Our chip design was a cylindrical-like structure that was cut in half and laid flat, thus creating two rectilinear chambers—one chamber for the hiPSC-Heps and one chamber for media to mimic the sinusoid. The chambers were separated by an isoporous polyethylene terephthalate (PET) membrane with 3 µm pores that acted as a diffusive barrier, mimicking the function of the liver sinusoidal endothelial cells that protect the hepatocytes from the shear stress forces of the blood flow. Prodanov et al. used a similar approach with pHeps and two additional cell lines to model contributions from endothelial cells and hepatic stellate cells (Prodanov et al., 2016). Our system used only hiPSC-Heps and relied on a different fluidic design to minimize shear stress within the hiPSC-Hep chamber, thereby reducing potential cell damage. Additionally, our liver MPS was designed to allow loading of a higher number of cells by centrifugation to improve 3D liver tissue formation and metabolic function.

Oxygen consumption was an important factor in designing the liver MPS since hepatocytes have particularly high oxygen consumption rates (OCRs) (Kidambi et al., 2009). The performance of the liver MPS was simulated and optimized in silico using computational modeling (Figure 2B; see *Material and Methods* for details). To maintain a high oxygen level in different flow conditions, the liver MPS was fabricated using a gas-permeable polydimethylsiloxane (PDMS). OCR in adult hepatocytes ranges from 0.3 to 0.9 nmol/s/10^6^ cells (Kidambi et al., 2009), which is an order of magnitude higher than ∼0.034 nmol/s/10^6^ in HepG2 cells (Provin et al., 2009; Mishra and Starly, 2009). The OCR in our hiPSC-Heps, measured using the Seahorse flux analyzer, was 0.1 nmol/s/10^6^ cells (Supplementary Figure S4), which is consistent with previously reported results (Mun et al., 2019). Using computational simulations, a media and cell chamber combination was designed that allowed for physiologically relevant oxygen levels in the cell chamber when considering the OCR of hiPSC-Heps and oxygen diffusion from the ambient through the PDMS walls. Using the OCR measured for our hiPSC-Heps, simulation showed a steady oxygen level of 0.16 mol/m^3^ across the device similar to the periportal region (pO2 0.10–0.12 mol/m^3^ or 60–70 mmHg) (Allen and Bhatia, 2003) after 24 h (Figure 2B, left). This tissue-level oxygen consumption is close to the physiological range and lower than hyperoxia (<160 mmHg) (Marconi et al., 2014). Additional simulation assuming no oxygen diffusion through the PDMS showed oxygen depletion in the device after 300 s due to the cells’ high OCR (Figure 2B, center). Shear stress, which is determined by the media flow rate used in the device, was lower than 5 dyn/cm^2^ previously found to diminish albumin and urea synthesis in rat pHeps (Park et al., 2005; Tilles et al., 2001). To give detail on the oxygen flux assumptions used in the simulation, additional COMSOL® modeling information on the mesh and computational domains of liver MPS used are provided in Supplementary Figure S5. In our optimal design, it took an estimated 300 s using a 20-μL/h flow rate for a small molecule to reach a uniform concentration inside an empty device accounting for diffusion of the drug across the membrane into the cell chamber (Figure 2B, right).

Fabrication of the liver MPS multilayer device was achieved with four parts: a glass slide, two layers of PDMS patterned with media and cell chambers, and a PET membrane. Media and cell chambers were permanently bonded into a device using oxygen plasma treatment and silanization for the PET membrane (Loskill et al., 2017) (Figure 2C), creating the final liver MPS (Figure 2D). The 3D liver microtissue in the MPS had a thickness of 80 μm covering the entire cell chamber of 5,560 μm (length) × 560 μm (width) in dimension. The liver MPS design permitted high cell viability after loading, indicated by live-dead staining of the cell chamber (Figure 2E). Cellular function in the device was further confirmed by visualization of nuclear morphology, density, and distribution of hiPSC-Heps, ALB and AFP expression, and formation of bile canaliculi (Figure 2F and Supplementary Figure S6). These assays showed that hiPSC-Heps were viable and maintained hepatocyte differentiation over 2 weeks of culture in the liver MPS.

### Comparison of Synthetic Function of Human Induced Pluripotent Stem Cell-Heps in Liver Microphysiological System and Conventional Cell Culture

**FIGURE 3:**
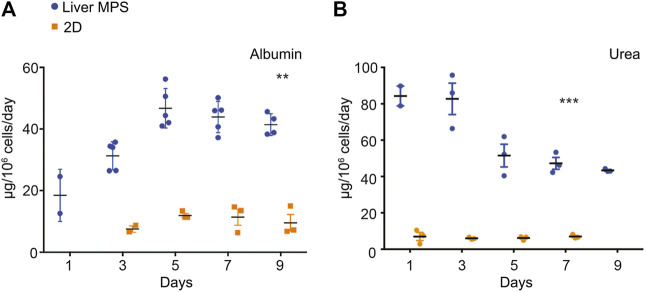
Synthetic function of hiPSC-Heps in conventional cell culture and liver MPS. **(A)** Albumin secretion into the media measured on the indicated days after seeding hiPSC-Heps into the liver MPS or replating them on conventional cell culture dishes (2D). Data are presented as the mean ± standard error of mean for five (liver MPS) and three (2D) independent experiments. Unpaired Student’s *t* test with equal standard deviation, **p < 0.05, ***p < 0.01. **(B)** Urea secretion into the media measured on the indicated days after seeding hiPSC-Heps into the liver MPS or replating them on conventional cell culture dishes. Data are presented as the mean ± standard error of mean for three independent experiments. Unpaired *t*-test with equal standard deviation, ***p < 0.01.

Potential differences in differentiation and function between hiPSC-Heps in the 3D environment of the liver MPS and under 2D conditions in conventional cell culture dishes were assessed by the analysis of albumin and urea secretion into the media. For this, hiPSC-Heps were released from the original differentiation cell cultures, split, and either seeded into devices or replated on cell culture dishes. At the end of a 9-day time course, hiPSC-Heps secreted 41.41 ± 3.53 µg/10^6^ cells/day albumin in the device, compared to 12.47 ± 0.53 µg/10^6^ cells/day in conventional culture (Student’s *t* test, *p* = 0.0036), a more than three-fold increase (Figure 3A). Similarly, hiPSC-Heps secreted 49.75 ± 5.04 µg/10^6^ cells/day urea in the device, compared to 7.37 ± 1.12 µg/10^6^ cells/day in conventional culture (Student’s *t* test, *p* = 0.0009), a seven-fold increase (Figure 3B). These results show that the liver MPS improves the synthetic function of hiPSC-Heps as compared with conventional cell culture.

### Comparison of Human Induced Pluripotent Stem Cell-Hep–Mediated Cisapride Metabolism in Liver Microphysiological System and Conventional Cell Culture

**FIGURE 4:**
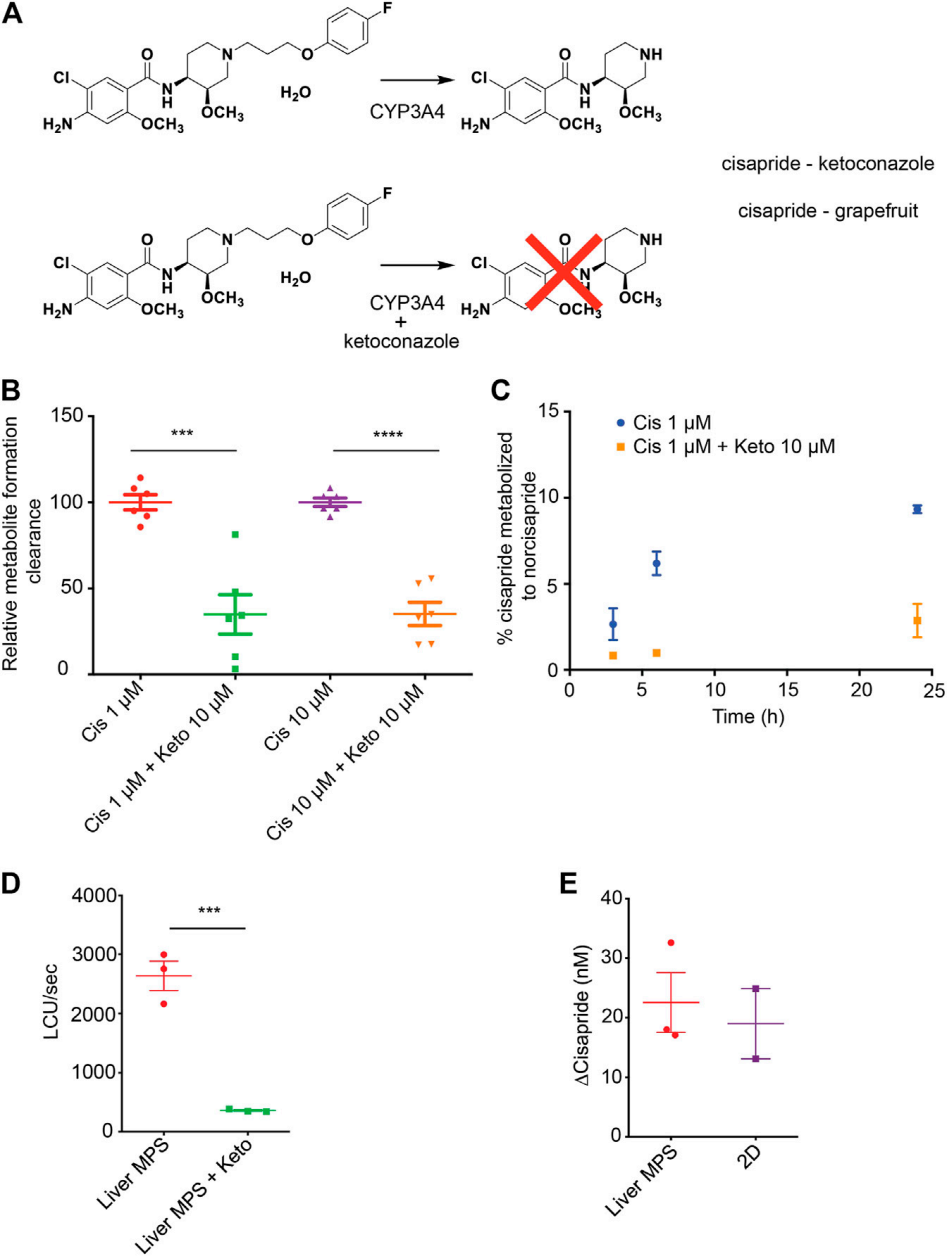
Cisapride metabolism in hiPSC-Heps in conventional cell culture and liver MPS. **(A)** Metabolism of the arrhythmogenic drug cisapride into norcisapride by CYP3A4 in the liver **(top)**. Drug–drug or drug–food interactions can inhibit this metabolic reaction. Ketoconazole (Keto) inhibits CYP3A4-driven metabolism of cisapride into norcisapride **(bottom)**. **(B)** Relative metabolite formation clearance for cisapride in hiPSC-Heps in conventional cell culture within 30 min. Data are presented as the mean ± standard deviation from three independent experiments with two biological replicates each. Unpaired Student’s *t* test, ***p < 0.0003, ****p < 0.0001. Experiments were performed in cell culture dishes with low drug absorption. **(C)** Percentage of cisapride metabolized to norcisapride with and without ketoconazole. Data are presented as the mean ± standard error of mean for two biological replicates of one experiment. The experiment was performed in cell culture dishes with low drug absorption. **(D)** CYP3A4 activity in the liver MPS measured with a luminescence assay with and without ketoconazole. Data are presented as the mean ± standard error of mean for three independent experiments. **(E)** Change in cisapride concentration in the liver MPS media channel and the supernatant of conventional cell cultures (2D). Data are presented as the mean ± standard error of mean for three independent experiments.

The arrhythmogenic drug cisapride is converted in pHeps into non-arrhythmogenic norcisapride ((Pearce et al., 2001) by CYP3A4 (Meuldermans et al., 1988; Bohets et al., 2000)) (Figure 4A). Therefore, the metabolism of cisapride can be inhibited or reduced by drugs and foods that act as CYP3A4 inhibitors such as ketoconazole or grapefruit (Figure 4A). Cisapride metabolism in hiPSC-Heps was assessed in conventional 2D cell culture using mass spectrometry to measure norcisapride formation within 30 min after the addition of 1 or 10 μM of the parental drug to the cells. Intra- and extracellular amounts of norcisapride were measured and put in relation to the cisapride input concentration and the time the cells were exposed to the drug, allowing the calculation of the metabolite formation clearance. Relative metabolite formation clearance for 1 μM of cisapride (100% +/− 10.81) was significantly reduced by the addition of 10 µM of ketoconazole to 34.87% +/− 28.04 (Figure 4B). Likewise, relative metabolite formation clearance for 10 μM of cisapride (100% +/− 6.08) was significantly reduced by the addition of 10 µM of ketoconazole to 35.18% +/− 16.55 (Figure 4B). To determine the ability of hiPSC-Heps to metabolize cisapride over an extended period of time, the percentage of cisapride converted into norcisapride was calculated 3, 6, and 24 h after adding 1 μM of cisapride to the cells—without CYP3A4 inhibition by 10 µM of ketoconazole, 2.66% ± 0.92, 6.19% ± 0.69, and 9.33% ± 0.23 of cisapride were metabolized, which dropped to 0.85% ± 0.02, 0.99% ± 0.06, and 2.87% ± 0.97 with ketoconazole, that is, a reduction by 68, 85, and 70%, respectively (Figure 4C).

Next, the hiPSC-Hep-mediated cisapride metabolism was analyzed in the liver MPS. First, a nondestructive luminescence assay (Promega P450-Glo; the same assay that was used in Figure 1F in 2D hiPSC-Heps)—allowing serial analysis of samples taken from the media channel—was used to assess CYP3A4 activity based on the cleavage-mediated activation of a luciferin substrate. As expected, additional infusion of 10 µM of ketoconazole into the device caused a seven-fold decrease in relative luminescence units (RLU) (2,640 ± 247 RLU vs. 361 ± 13 RLU; Student’s *t* test, *p* = 0.0008) (Figure 4D). Second, mass spectrometry was used to compare cisapride metabolism between hiPSC-Heps in the liver MPS or in conventional cell culture, which showed that cisapride concentrations were similarly reduced under both culture conditions 12 h after exposure to 100 nM of the drug (Figure 4E). These results establish that the small media volumes of the liver MPS are sufficient for luminescence assays and mass spectrometry, and that CYP3A4-mediated cisapride metabolism is maintained in hiPSC-Heps transferred into the device.

### Drug–Drug Interaction Studies in Integrated Liver and Cardiac Microphysiological Systems

**FIGURE 5:**
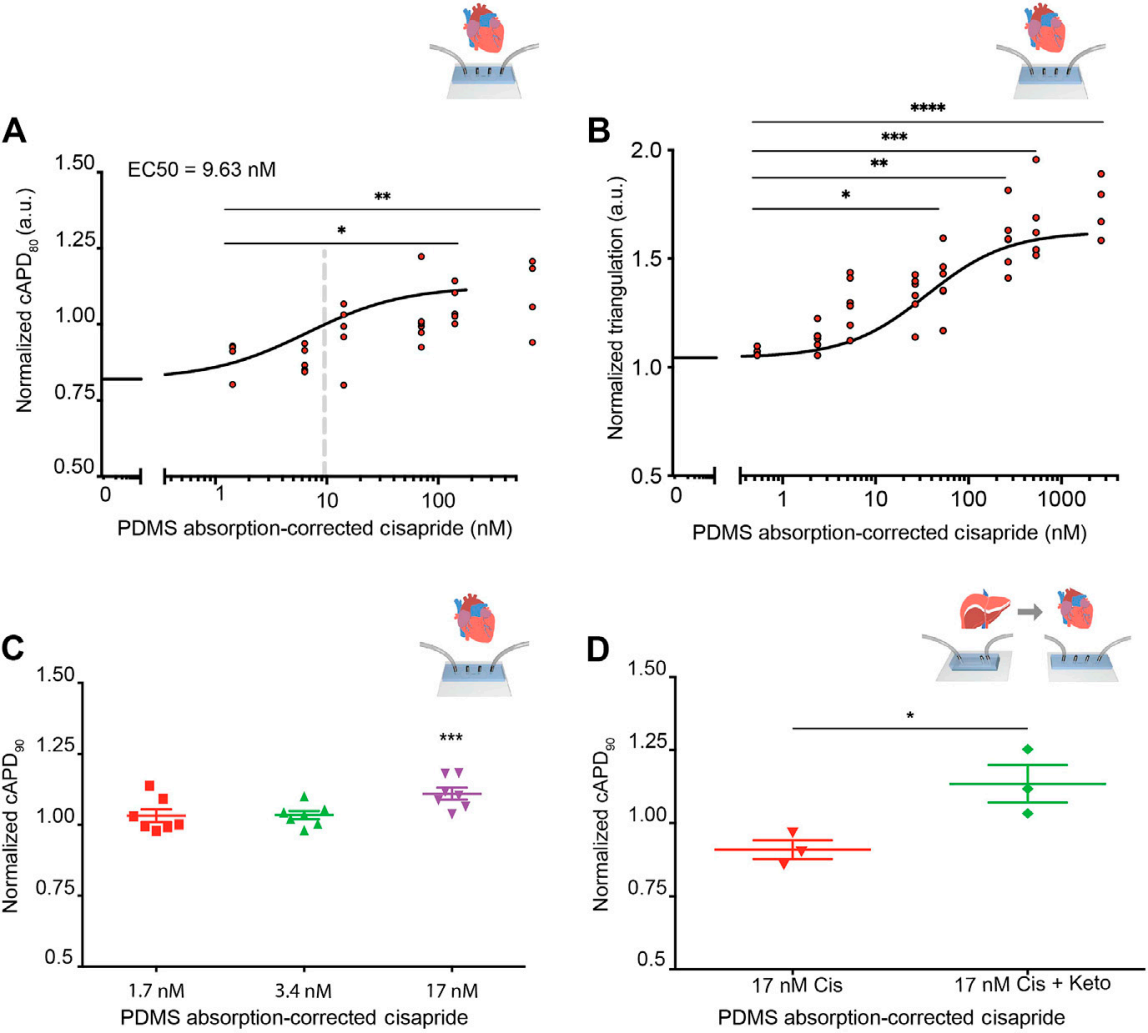
Cisapride effects on cardiac MPS and DDI in integrated liver and cardiac MPSs. **(A)** Cisapride-induced changes in cAPD_80_ in the cardiac MPS. Nonlinear regression fit log(inhibitor) vs. response (Hill Equation using three parameters) was used to obtain EC_50_ value (9.63 nM, gray vertical line). Cisapride values were corrected for PDMS drug absorption of 64%. Statistical differences are based on one-way ANOVA (*p* values: * = 0.0259 and ** = 0.0095). **(B)** Cisapride-induced changes in beat shape plotted as triangulation value (cAPD_80_ - cAPD_30_)/cAPD_80_. Cisapride values are corrected for PDMS drug absorption of 64%. Statistical differences are based on one-way ANOVA (*p* values: * = 0.0174, ** = 0.048, *** = 0.0004 and **** <0.0001). **(C)** Effect of increasing concentrations of cisapride on cAPD_90_ in the cardiac MPS. The cAPD_90_ values of the treatments were normalized to the respective values for vehicle controls. Data are presented as the mean ± standard error of mean for at least six independent experiments. Ordinary one-way ANOVA with Dunnett’s correction for multiple comparisons test, ***p < 0.0001. Cisapride values are corrected for PDMS drug absorption of 64%. **(D)** Effect of cisapride metabolized in liver MPS on cAPD_90_ in cardiac MPS. cAPD_90_ values of the treatments were normalized to the 0 nM control values. Data are presented as the mean ± standard error of mean for three experiments. Unpaired Student’s *t* test with equal standard deviation was used, *p < 0.05. Cisapride values are corrected for PDMS drug absorption of 64%.

Before integration of liver MPS and cardiac MPS, cisapride-induced changes in beat rate and electrophysiology were analyzed in the cardiac MPS, which was created as previously described, including cardiomyocyte generation from the same hiPSC line used to generate hiPSC-Heps (Mathur et al., 2015; Huebsch et al., 2018). In the clinical setting, the QT interval is used as a measure of cardiac electrophysiology. Since the QT interval depends largely on the heart rate, it is typically reported as QTc, a value normalized for 60 beats per minute (BPM) using Fridericia’s formula to introduce the heart rate correction (Fridericia, 1920; Fridericia, 2003). Using similarly corrected action potential duration at 80% of the repolarization (cAPD_80_) as a surrogate for QTc, an EC_50_ = 9.63 nM was observed in the cardiac MPS for cisapride concentrations ranging from 0 to 180 nM (after the correction for absorption into the PDMS of the device) (Figure 5A). Beyond 180 nM, data became dispersed and eventually cAPD_80_ values decreased, indicating toxic effects at the highest drug doses (Supplementary Figure S7A). We also investigated triangulation, a metric that characterizes disturbances of the repolarization and more accurately identifies proarrhythmic substrates (Hondeghem, 2008). An increase in the triangulation metric, which we calculated as (cAPD_80_–cAPD_30_)/cAPD_80_, indicated a shift from rectangular to more triangular beat shape, associated with arrhythmia risks (Hondeghem et al., 2001). We found a strong dose-dependent increase in triangulation values in response to cisapride with significant changes at 36 nM and higher (Figure 5B). Exposure of the cardiac MPS to 17 nM of cisapride caused a statistically significant prolongation of the cAPD (Figure 5C). Beat rate was significantly elevated only at extremely high cisapride concentrations (Supplementary Figure S7B).

To facilitate the DDI study of cisapride and ketoconazole, and its effects on cardiac function, the liver and cardiac MPSs were integrated by functional coupling. A prerequisite was to use a common culture medium that supported the function of both hiPSC-Heps and hiPSC-derived cardiomyocytes in their respective devices. Literature reports on common media compositions for liver and cardiac tissues vary greatly (Oleaga et al., 2018; Zhang et al., 2017; Oleaga et al., 2016) and typically suitability for both tissues is verified by simple viability assays. In our work, we used the liver media for drug testing, which ensured ideal and consistent conditions for all liver data. To verify compatibility with the cardiac chip, we demonstrated that the beat rate was not altered in response to the common media, which was a more stringent and functionally relevant criterion than cell viability. Baseline beat rate was indistinguishable in RPMI plus B27-supplement or after a 30-min incubation in Williams’ E + Cocktail B media (Supplementary Figure S8). For DDI studies, cardiac MPSs were incubated with Williams’ E + Cocktail B media for 30 min and baseline cAPD_90_ values were recorded and normalized for each device. Next, the efflux from the liver MPS was introduced into the cardiac MPS via its media ports. The cAPD_90_ in the cardiac MPSs was not altered by efflux media from the liver MPSs perfused with 17 nM of cisapride for 8 h. However, efflux media from liver MPSs perfused with 17 nM cisapride together with 10 µM of ketoconazole for 8 h significantly prolonged the cAPD_90_ in cardiac MPSs (Figure 5D). These results reproduce with functionally coupled hiPSC-derived liver and cardiac MPSs *in vitro* the clinically observed DDI between cisapride and ketoconazole (Michalets and Williams, 2000; Wysowski et al., 2001; Paakkari, 2002; Shah, 2002).

## Discussion

This work focused on developing a functional liver MPS constructed from hiPSC-Heps, and using the system to study DDI-mediated inter-organ toxicity in a liver—cardiac MPS. There are two major reasons for creating better *in vitro* models of human hepatotoxicity and liver diseases that address the “fit for purpose” criteria used in the pharmaceutical industry. First, earlier and accurate safety profiling of a larger number of compounds, but still high throughput, can eliminate false-positive attrition. The second is for testing of more challenging hepatotoxicity mechanisms and complex disease models over longer time periods with high content models representing physiologically relevant fluid flow and cell types (Hewitt et al., 2007; Song et al., 2009; Godoy et al., 2013; Soldatow et al., 2013). Focusing on the latter, we created a liver MPS employing hiPSC-Heps that provides a virtually unlimited source of patient-specific cells. In-depth characterization of hiPSC-Heps generated with the optimized differentiation protocol showed that levels of drug uptake, metabolism, and efflux were inferior to pHeps, but sufficient for use in drug screening and toxicity studies.

Microfabrication and microfluidic technologies provide a microenvironment that resembles *in vivo* conditions more closely than conventional cell culture (Dittrich and Manz, 2006; Sung and Shuler, 2010). For example, in multiwell plates and culture flasks hepatocytes are cultured in monolayers with excessive medium volume-to-cell number ratios. More physiological medium-to-cell ratios can be achieved by using the small system volumes of microfluidic devices (Khetani and Bhatia, 2008; Dash et al., 2009), which preserves the efficacy of low-concentration autocrine and paracrine signals, including secreted molecules, growth factors, matrices, and hormones (Abhyankar et al., 2006). Along these lines, the microfabricated liver MPS described in this study can be used under a controlled range of flow rates, providing flexibility for optimizing cell viability and function. Using a PET membrane to mimic an endothelial cell barrier, we protected the cells from flow-induced shear stress, which can compromise viability (Tilles et al., 2001; Park et al., 2008; Vinci et al., 2011). Moreover, under low shear stress conditions, perfusion/flow cultures provide better function of hepatocytes than static cultures, presumably due to greater nutrient exchange (Albert et al., 2007). Other factors promoting function of hiPSC-Heps in the device include direct 3D cell–cell contact and the low ratio of medium-to-cell volume. Whether a small subset of hiPSCs not committing to hepatocyte differentiation, but giving rise to mesenchymal cell types instead (data not shown), further aided hiPSC-Hep function remains to be determined (Bhatia et al., 1999). Currently, cross-laboratory studies are ongoing for performance evaluation of our MPSs compared with other hiPSC-based models.

Our liver–cardiac MPS successfully replicated a highly publicized example of DDI, severely impacting human health despite successful clinical trials. Cisapride, a prokinetic used to treat gastroesophageal reflux (Wiseman and Faulds, 1994), caused prolongation of the QT interval, ventricular arrhythmias, and torsade de pointes (Wysowski and Bacsanyi, 1996; Wysowski et al., 2001), particularly if metabolism of the drug was impaired (Wiseman and Faulds, 1994; Shah, 2002). In humans, cisapride is extensively metabolized to inactive norcisapride through N-dealkylation (41–45% of the administered dose) and to several minor metabolites (Meuldermans et al., 1988). CYP3A4 is involved in the metabolism of most drugs in the human liver, including cisapride N-dealkylation (Shimada et al., 1996; Pearce et al., 2001; Hyland et al., 2003). By 2007, DDI with ketoconazole leading to inhibition of CYP3A4 or other risk factors caused 300 deaths and 16,000 cardiac injuries, ultimately leading to the voluntary removal of cisapride from the market (Comeau, 2007).

We used this example to demonstrate the robustness of liver-cardiac MPS interaction studies to predict DDI. First, we validated the cisapride metabolism of hiPSC-Heps using LC-MS/MS and a luminescence assay and confirmed that the CYP3A4 metabolic function was inhibited by the addition of ketoconazole to the media using the norcisapride metabolite as a reference value (Supplementary Figure S9). Using the cAPD as a proxy for the QTc interval, we confirmed cisapride-induced cAPD prolongation in the cardiac MPS in a dose escalation manner, and identified an EC_50_ of 9.63 nM. This EC_50_ value is more sensitive than the previous estimates of 32 nM (Liang et al., 2013) in 2D hiPSC-derived cardiomyocytes and 21.2 nM (Haraguchi et al., 2015) or 20 nM (Crumb et al., 2016) in human HEK293 cells (Supplementary Table S1). Our investigation of arrhythmia using triangulation as a metric correlates with the observed cAPD increase and underlines the arrhythmogenic risk associated with cisapride. The fact that triangulation values increase over the entire dose range while cAPD_80_ values drop at the highest (toxic) doses further underlines the robustness of triangulation as a metric for arrhythmogenic risk.

Based on the estimated therapeutic plasma concentration (ETPC) of cisapride (2.6–4.9 nM) (Redfern et al., 2003), we calculated a margin of safety of approximately 3.7-fold, which is close to the clinically determined safety margin of 1.1- to 6.5-fold (Redfern et al., 2003; Liu et al., 2006; Harris et al., 2013). Redfern et al. (2003) have suggested that a margin of 30-fold provides a degree of safety necessary for drugs that can cause cardiac arrhythmias. Next, using functionally coupled liver and cardiac MPSs, prolongation of cAPD was eliminated when cisapride was passed through the liver MPS before exposure to the cardiac MPS. However, when the cardiac MPS was exposed to media from the liver MPS in the presence of cisapride and ketoconazole, prolongation of cADP was again observed, demonstrating the inhibitory effect ketoconazole has on the liver metabolism of cisapride. Collectively, these data demonstrate that the liver–cardiac MPS would have correctly predicted cisapride’s toxic effects in the presence of ketoconazole, potentially saving lives by indicating the need for appropriate DDI warning.

The ability to predict DDI validates the efficacy of the functionally coupled liver and cardiac MPSs, and illustrates how the system can identify unforeseen DDI-induced complications, albeit in this work retrospectively. The integrated system exhibits a level of biological complexity that starts to approach *in vivo* conditions, thereby providing a powerful platform for simultaneously screening drug efficacy and toxicity. Although differentiation and function of hiPSC derivatives generated with current protocols are not equal to their primary counterparts, our study shows that in-depth functional characterization makes it possible to use these cells to build genetically defined faithful multiorgan models. Furthermore, hiPSC technology allows generation of multiorgan human disease models not attainable with animals, opening a path for more accurate drug efficacy analysis in real populations.

## Data Availability

The raw data supporting the conclusions of this article will be made available by the authors, without undue reservation.
