# Supplementary material for: Integrated Isogenic Human Induced Pluripotent Stem Cell–Based Liver and Heart Microphysiological Systems Predict Unsafe Drug–Drug Interaction
Source: Front Pharmacol. 2021 May 7;12:667010. doi: 10.3389/fphar.2021.667010 (PMC8138446; doi:10.3389/fphar.2021.667010)
Supplement: Supplementary file 1 [file DataSheet1.pdf]

Supplementary Material

Supplementary Table 1. Cisapride EC_{50} values compiled from the literature.

The estimated therapeutic plasma concentration (ETPC) of cisapride is 2-5 nM. MEA: multielectrode array; hiPSC-CMs: human induced pluripotent stem cell-derived cardiomyocytes.

Drug	Description of effect	2D or 3D	Cell type/ Study model	Analysis	Ion Channel	EC_{50} (nM)	Reference
Cisapride	5-HT4 receptor agonist	3D	hiPSC-CMs	Voltage dye	hERG	9.6	This study
Cisapride	5-HT4 receptor agonist	2D	CHO, hERG-transfected	Patch clamp	hERG	36.5	(Kim et al., 2017)
Cisapride	5-HT4 receptor agonist	2D	HEK-293	Patch clamp	hERG	21.2	(Haraguchi et al., 2015)
Cisapride	5-HT4 receptor agonist	2D	HEK-293	Patch clamp	hERG	10	(Crumb et al., 2016)
Cisapride	5-HT4 receptor agonist	2D	hiPSC-CMs	MEA	hERG	20	(Redfern et al., 2003), (Kramer et al., 2013), (Ando et al., 2017)
Cisapride	5-HT4 receptor agonist	2D	HEK-293	Patch clamp	hERG	20-45	(Redfern et al., 2003)
Cisapride	5-HT4 receptor agonist	2D	HEK-293	Patch clamp	hERG	20	(Kramer et al., 2013)
Cisapride	5-HT4 receptor agonist	3D spheroid	hiPSC-CMs	Calcium oscillation	hERG	202	(Sirenko et al., 2017)
Cisapride	5-HT4 receptor agonist	2D	hiPSC-CMs	Calcium oscillation	hERG	11	(Sirenko et al., 2017)
Cisapride	5-HT4 receptor agonist	2D	hiPSC-CMs	Patch clamp	hERG	32	(Sirenko et al., 2013)
Cisapride	5-HT4 receptor agonist	2D	HEK-293	Patch clamp	hERG	27	(Kirsch et al., 2004), (Wible et al., 2005)
Cisapride	5-HT4 receptor agonist	2D	COS-7	Patch clamp	hERG	240	(Potet et al., 2001)
Cisapride	5-HT4 receptor agonist	2D	HEK-293	Patch clamp	hERG	6.5	(Mohammad et al., 1997)
Cisapride	5-HT4 receptor agonist	2D	HEK-293	Patch clamp	hERG	14.4	(Wang et al., 2003)
Cisapride	5-HT4 receptor agonist	2D	hiPSC-CMs	Patch clamp	hERG	32-35	(Liang et al., 2013)
Cisapride	5-HT4 receptor agonist	2D	HEK-293	Patch clamp	hERG	27	(Wible et al., 2005)
Cisapride	5-HT4 receptor agonist	2D	HEK-293	Patch clamp	hERG	44.5	(Rampe et al., 1997)
Cisapride	5-HT4 receptor agonist	2D	CHO-K1	Patch clamp	hERG	23.6	(Walker et al., 1999)
Cisapride	5-HT4 receptor agonist	2D	HEK-293	Patch clamp	hERG	18	(Martin et al., 2004)
Cisapride	5-HT4 receptor agonist	2D	HEK-293	Patch clamp	hERG	30	(Furuta et al., 2004)
Cisapride	5-HT4 receptor agonist	2D	HEK-293	Patch clamp	hERG	15	(Fossa et al., 2004)

frontiers

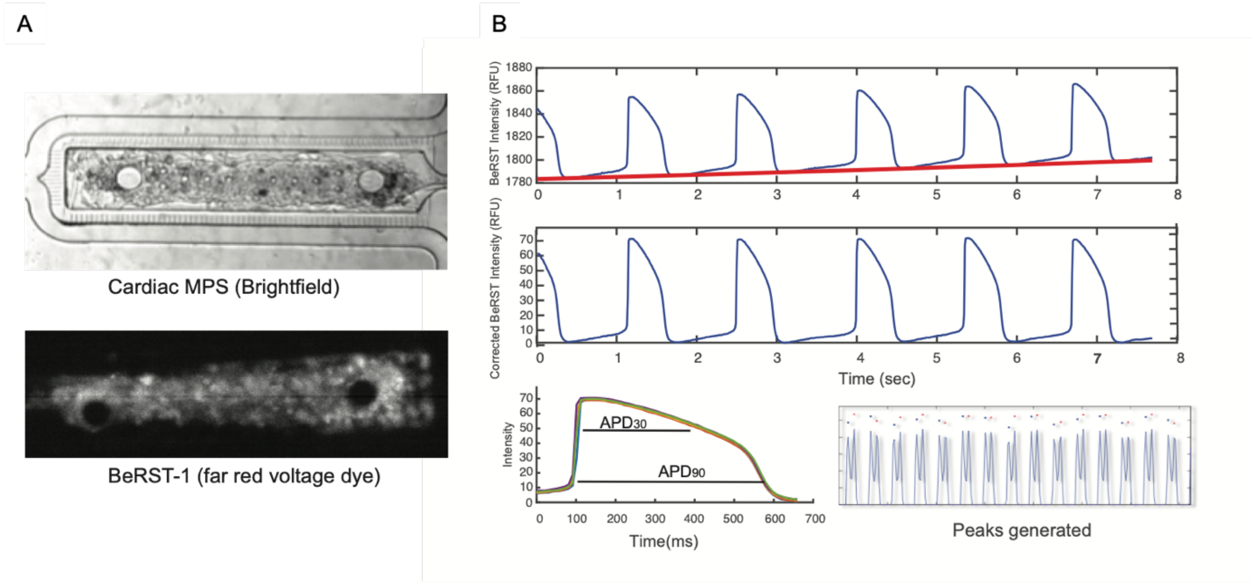

Supplementary Figure 1. Voltage-sensitive dye BeRST-1 and MATLAB motion tracking analysis software. (A) Cardiac MPS brightfield image (top) and labeling with far red voltage dye BeRST-1 (bottom). (B) Data sample of quantification of the beat rate and APD signal. BeRST-1 videos were analyzed for beat rate and beat duration at 30%, 80%, and 90% peak height (APD_{30}, APD_{80}, and APD_{90}) using custom MATLAB and Python code.

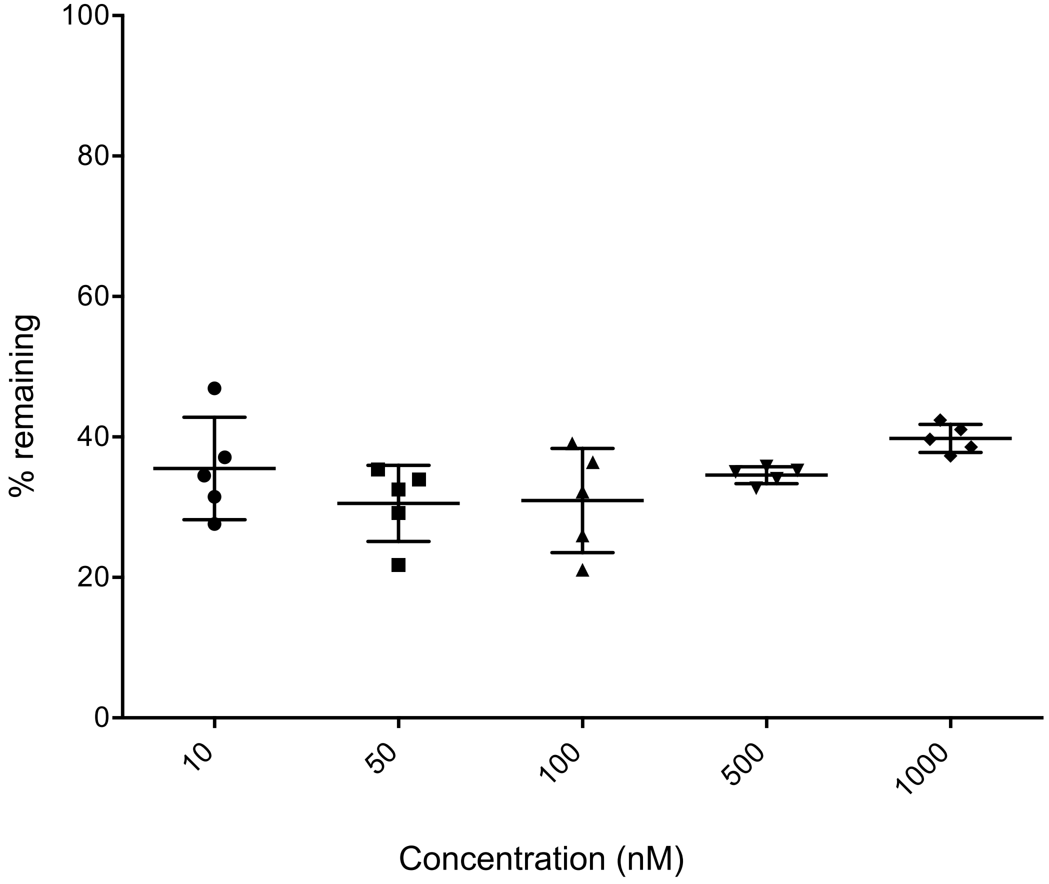

Supplementary Figure 2. Mass spectrometry analysis of cisapride absorption in PDMS. Absorption of different concentrations of cisapride in PDMS in the liver MPS was analyzed to correct the concentrations used in the experiments. Ion abundances of the drug in stock samples and in efflux media was measured using LC-MS/MS and % remaining was calculated. One device was used per cisapride concentration of 10, 50, 100, 500, and 1,000 nM.

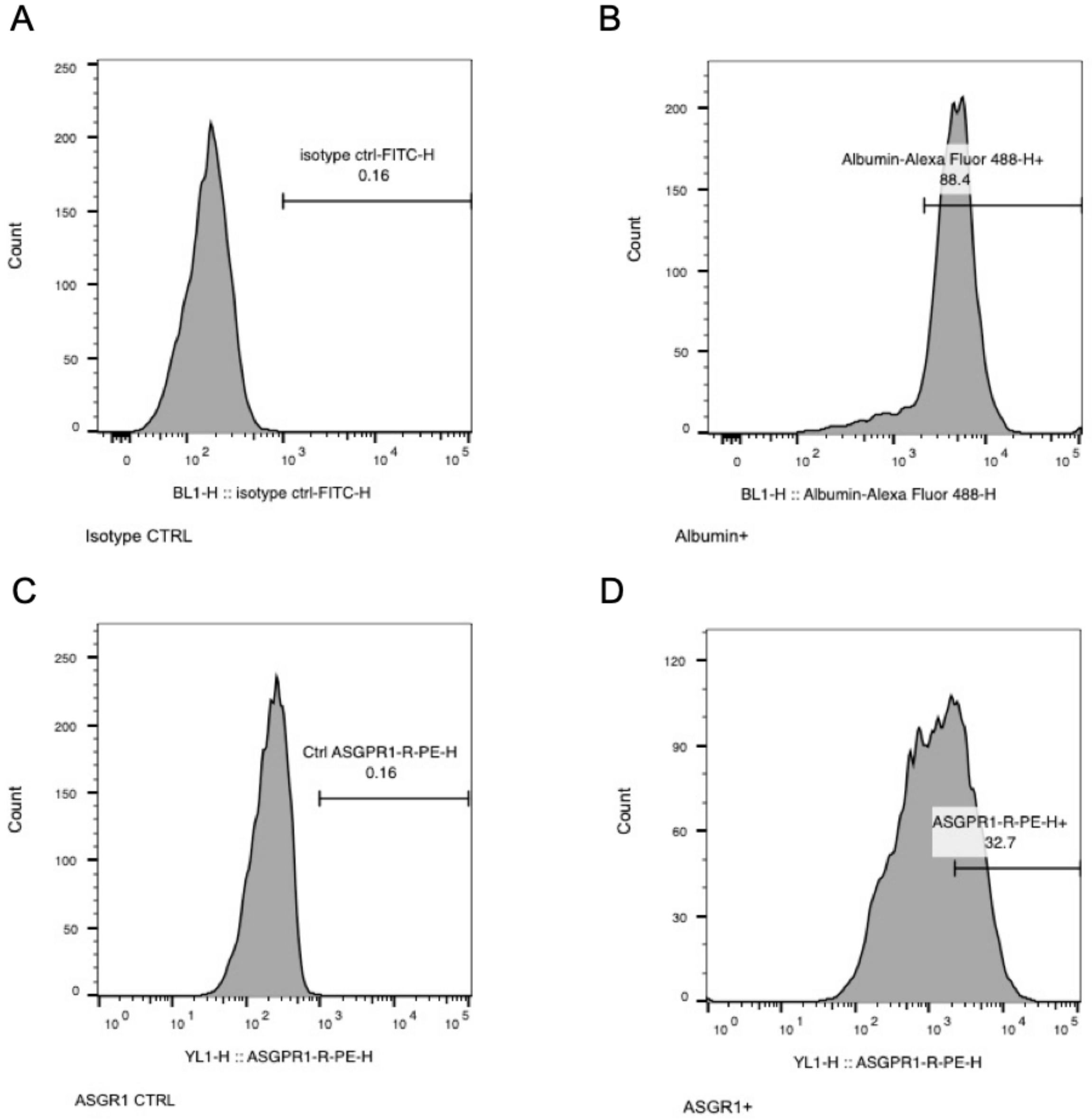

Supplementary Figure 3. Flow cytometry analysis of hiPSC-Heps. Representative flow cytometry results of hiPSC-Heps at day 23 of the differentiation protocol. Isotype control FITC (A), albumin-positive cells (B), ASGR1 control (C) and ASGR1-positive cells (D). Numbers indicate % positive cells.

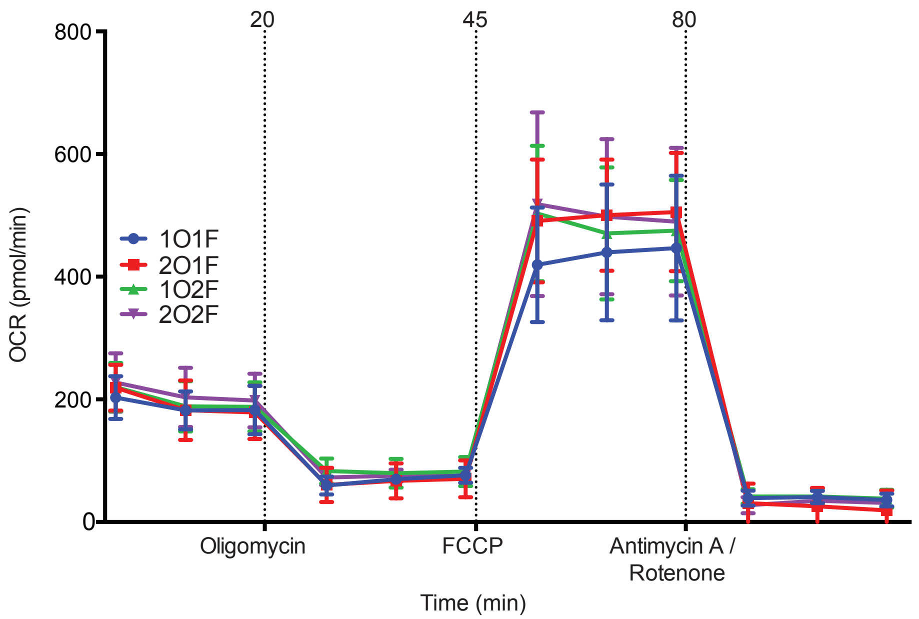

Supplementary Figure 4. Oxygen consumption rate (OCR) of hiPSC-Heps. Direct measurement of OCR was made by sequential exposure of hiPSC-Heps to 1 µM oligomycin (1O1F and 1O2F) or 2 µM oligomycin (2O1F and 2O2F), 1 and 2 µM carbonyl cyanide-4-(trifluoromethoxy)phenylhydrazone (FCCP) and rotenone/antimycin A. Basal respiration was measured for 20 min. Oligomycin was injected at 20 min to block ATP synthase (complex V), thereby leading to a decrease in electron flow through the electron transfer chain (ETC) and the observed decrease in OCR. FCCP is an uncoupling agent disrupting the mitochondrial membrane potential. FCCP was injected at 50 min and its disinhibition of the electron flow through the ETC led to the determined maximal OCR. At 80 min a combination of rotenone (complex I inhibitor) and antimycin A (complex III inhibitor) was injected to shut down mitochondrial respiration.

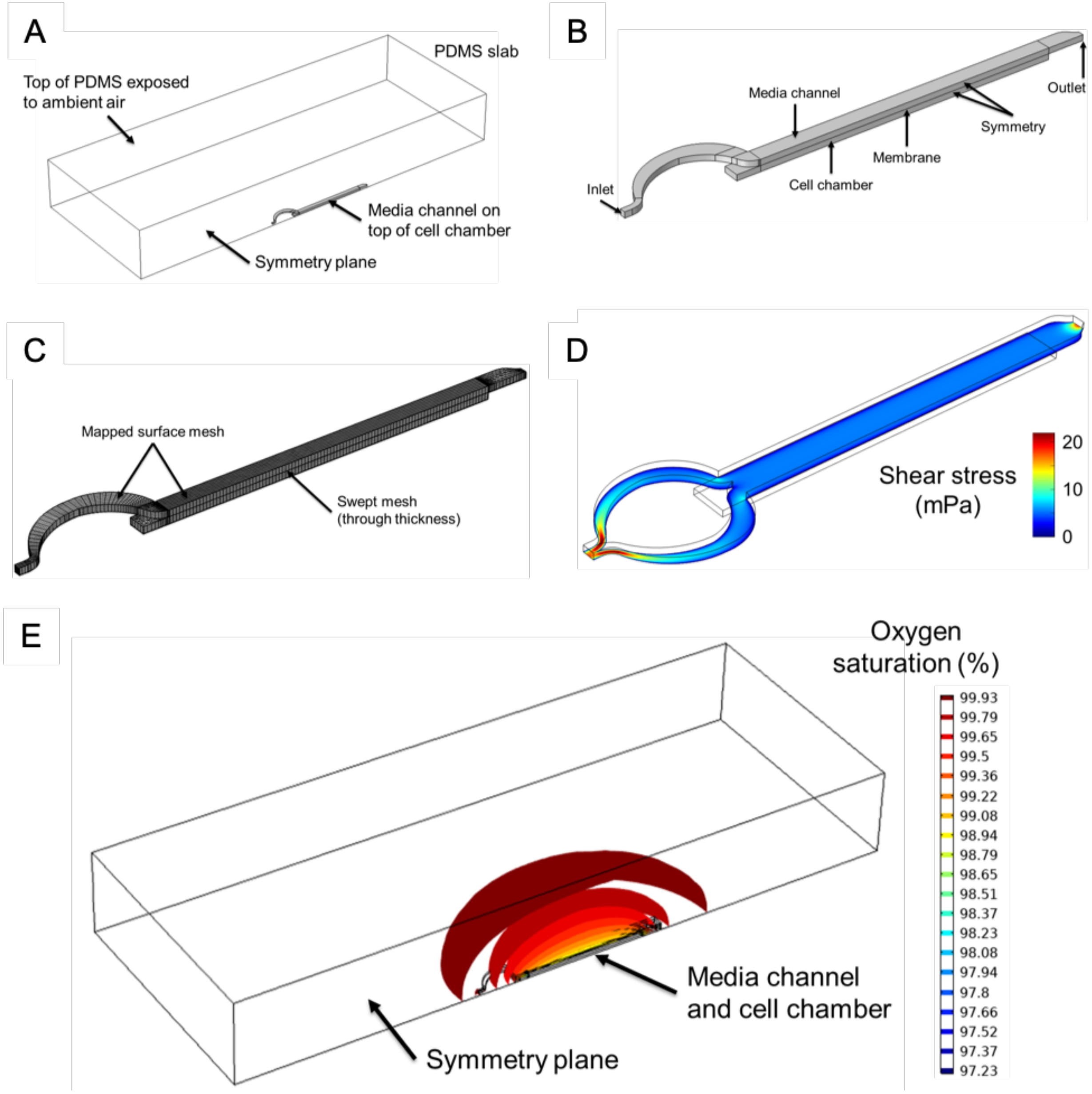

Supplementary Figure 5. Liver MPS COMSOL Multiphysics® model setup and results. Liver MPS COMSOL Multiphysics® model setup (A), liver MPS model setup of cell chamber and media channel (B), liver MPS model mesh of cell chamber and media channel (C), shear stress on membrane (media channel side) at 20 µL/h (D), and oxygen saturation in PDMS at 24 h (E). The O_2 concentrations in the PDMS, and in particular at the media channel and cell chamber walls, are nearly saturated (i.e., above 97% of ambient O_2 partial pressure).

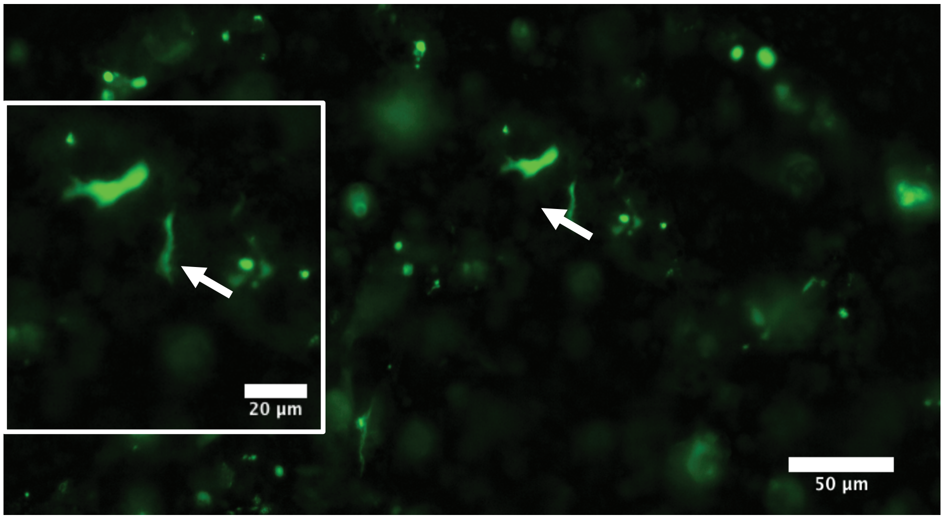

Supplementary Figure 6. Stable differentiation and function of hiPSC-Heps in liver MPS. hiPSC-Heps form bile canaliculi after 2 weeks in culture inside the liver MPS as shown by MRP2-dependent intracanalicular transport and accumulation of the metabolite glutathione methylfluorescein 1h after incubation with 5-chloromethylfluorescein diacetate.

A

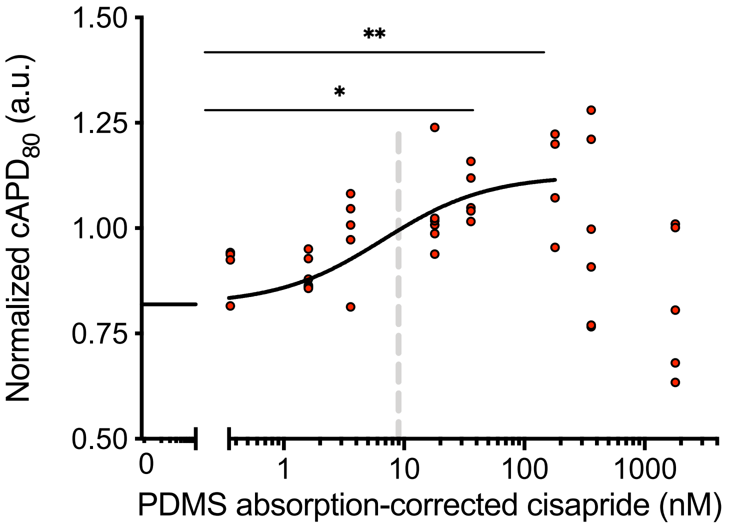

B

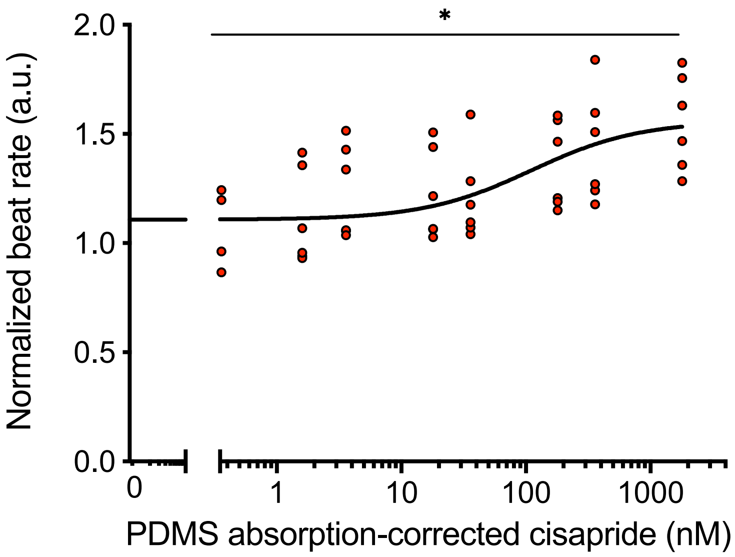

Supplementary Figure 7. Cisapride dose escalation study in the cardiac MPS. (A) Normalized cADP80 data were over the full range of concentrations tested. Data at the highest doses were highly variant due to drug toxicity and cessation of beating. Data were fit as described for Figure 5A in the main text. (B) Spontaneous beat rate over a dose range of 0 to 1.8 mM is shown. Cisapride values are corrected for 64% PDMS absorption. Statistical differences are based on one-way ANOVA (*p* value: * = 0.0174).

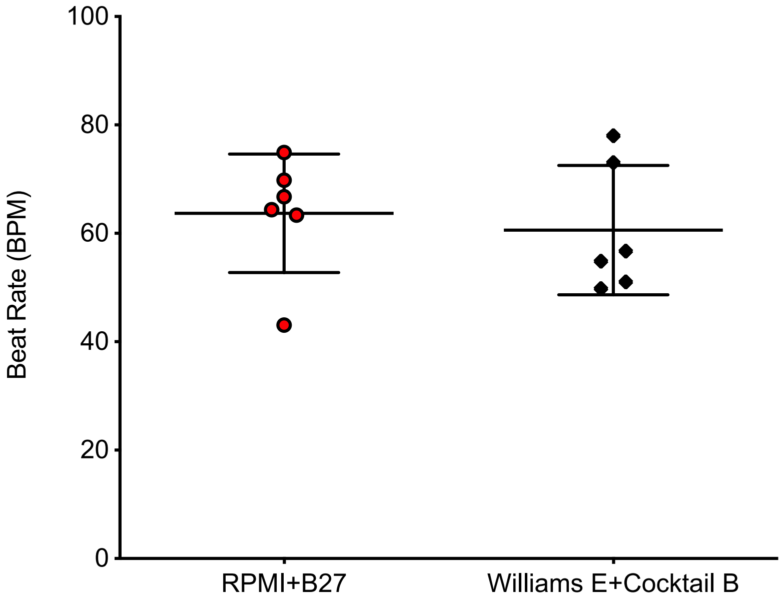

Supplementary Figure 8. Effect of different media on beat rate in the cardiac MPS. Effects of different culture media on spontaneous beat rate of hiPSC-CMs in cardiac MPS.

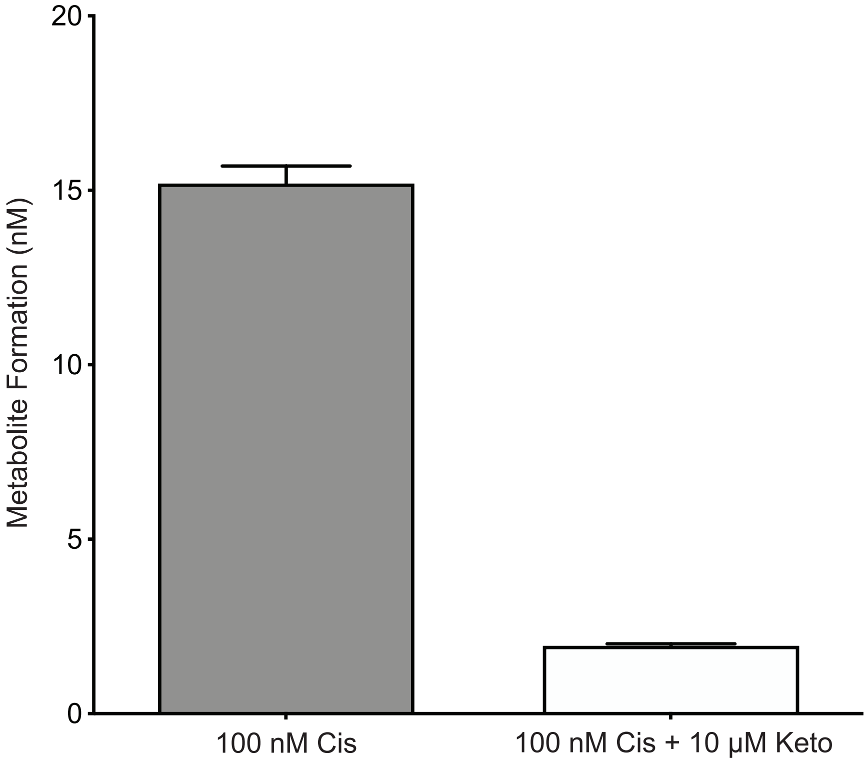

Supplementary Figure 9. Characterization of low-dose cisapride metabolism in hiPSC-Heps in conventional culture. Mass spectroscopy analysis of hiPSC-Heps using UFLC-MS/MS shows CYP3A4-mediated cisapride-to-norcisapride metabolism at 100 nM drug input and inhibition by ketoconazole.

Supplemental Methods

Bile Efflux Testing

hiPSC-Heps were tested for normal canalicular efflux of CMFDA at day 7. CMFDA (Life Technologies) was prepared at 4 µM in HCM and added to the liver MPS. The devices were removed from the perfusion pump and imaging was performed using a Nikon Eclipse microscope for collection of 488/525 nm (ex/em) after 1-hour exposure with 30-min intervals over 1 h.

Oxygen Consumption Rate

Oxygen consumption was analyzed in hiPSC-Heps (on day 25 of differentiation) with the Seahorse XF24 cellular respirometer (Agilent). Mitochondrial stress tests were performed on hiPSC-Heps at 100% confluence (80,000 cells/well) in V28 microplates, with XF assay medium supplemented with 1 mM pyruvate (Gibco), 2 mM glutamine (Gibco), and 25 mM glucose (Sigma) at pH 7.4 and sequential additions via injection ports of oligomycin (1 and 2 µM final), FCCP (1 and 2 µM final), and antimycin A/rotenone (1 µM final) during respirometry (concentrated stock solutions solubilized in 100% ethanol [2.5 mM] for mitochondrial stress test compounds). Plates were incubated in a CO_2-free incubator at 37°C for 1 h to allow temperature and pH equilibration.

Liver MPS COMSOL Multiphysics® model

The liver MPS COMSOL® model comprising PDMS slab containing media channel and cell chamber separated by a porous PET membrane (**Supplementary Fig. 3A)**. By symmetry about the vertical center plane, the computational domain was half the physical domain. The cell chamber base was flush with base of 3.5 mm thick PDMS slab. The top of the PDMS slab was exposed to ambient air with specified oxygen (O_2) concentration. The base of the device (base of cell chamber and PDMS) was assumed O_2 impermeable. The O_2 flux through the PDMS slab's vertical sides was neglected, which was a good assumption since these sides were sufficiently far away from the media channel and cell chamber. The liver MPS model setup of cell chamber, media channel, and membrane portions of computational domain are shown in **Supplementary Fig. 3B**. Media channel and cell chamber heights were 100 µm and the membrane thickness was 15 µm. Flow enters the inlet at 20 µL/h and exits at the outlet. Laminar inflow and outflow conditions were used in COMSOL®. The membrane was modeled as a 2D planar surface between the media channel and cell chamber, with effective diffusivities for O_2 and small molecules. Fluid flow across the membrane was neglected, and zero flow velocity was assumed throughout the cell chamber. No-slip conditions were imposed at all walls and membrane surfaces within the medial channel. For O_2 transport simulations, cells were assumed to fill the cell chamber volume at a specified density and consume O_2 at a specified rate (see main text for details). Liver MPS model mesh of the cell chamber, media channel, and membrane portions of computational domain are shown in **Supplementary Fig. 3C**. Mapped meshes were used to control the axial and lateral mesh resolutions on the straight and curved portions of the media channel. Triangular mesh elements were used at connection points, and surface meshes were then swept downward through the thicknesses of the media channel and cell chamber (5 elements through each). Mesh sensitivity analysis was performed to show that this mesh resolution yields sufficiently accurate numerical results. Quadratic finite elements were used for the flow velocity components and species concentration, while linear elements were used for fluid pressure. For shear stress calculations on the membrane (media channel side) at 20 µL/h, no-slip conditions were assumed at all walls as well as along the membrane

(**Supplementary Fig. 3D**). O_2 saturation in PDMS was determined at 24 h for a flow rate of 20 µL/h (**Supplementary Fig. 3E**).

Cardiac MPS preparation

The cardiac MPS was prepared as described in detail in a separate publication(Mathur et al., 2015;Huebsch et al., 2020). In brief, standard soft lithography was used to prepare the microfluidic devices from PDMS casted onto patterned SU-8 wafers. PDMS blocks featuring the microchannels were permanently bonded to glass substrates after oxygen plasma surface activation (24s, 21W, 0.59 Torr).

hiPSCs were differentiated into cardiomyocytes and isogenic stromal cells using previously published protocols(Huebsch et al., 2020). In order to mimic the native cellular composition of the human heart(Naito et al., 2006), 80% hiPSC-derived cardiomyocytes were mixed with 20% hiPSC-derived stromal cells and loaded into the cell chambers (20,000 cells/chamber). The cardiac MPS was cultured in RPMI 1640 medium (11875-093; Gibco) supplemented with 2% B-27 (17504-044; Gibco). A detailed characterization of the cells and microtissues can be found in a separate study(Huebsch et al., 2020).

References

Ando, H., Yoshinaga, T., Yamamoto, W., Asakura, K., Uda, T., Taniguchi, T., Ojima, A., Shinkyo, R., Kikuchi, K., and Osada, T. (2017). A new paradigm for drug-induced torsadogenic risk assessment using human iPS cell-derived cardiomyocytes. Journal of pharmacological and toxicological methods 84, 111-127.

Crumb, W.J., Vicente, J., Johannesen, L., and Strauss, D.G. (2016). An evaluation of 30 clinical drugs against the comprehensive in vitro proarrhythmia assay (CiPA) proposed ion channel panel. Journal of pharmacological and toxicological methods 81, 251-262.

Fossa, A.A., Wisialowski, T., Wolfgang, E., Wang, E., Avery, M., Raunig, D.L., and Fermini, B. (2004). Differential effect of HERG blocking agents on cardiac electrical alternans in the guinea pig. European journal of pharmacology 486, 209-221.

Furuta, S., Kamada, E., Omata, T., Sugimoto, T., Kawabata, Y., Yonezawa, K., Wu, X.C., and Kurimoto, T. (2004). Drug–drug interactions of Z-338, a novel gastroprokinetic agent, with terfenadine, comparison with cisapride, and involvement of UGT1A9 and 1A8 in the human metabolism of Z-338. European journal of pharmacology 497, 223-231.

Haraguchi, Y., Ohtsuki, A., Oka, T., and Shimizu, T. (2015). Electrophysiological analysis of mammalian cells expressing hERG using automated 384-well-patch-clamp. BMC Pharmacology and Toxicology 16, 16-39.

Huebsch, N., Charrez, B., Siemons, B., Boggess, S.C., Wall, S., Neiman, G., Charwat, V., Jæger, K.H., Cleres, D., Telle, Å., Lee-Montiel, F.T., Jeffreys, N.C., Deveshwar, N., Edwards, A., Serrano, J., Snuderl, M., Stahl, A., Tveito, A., Miller, E.W., and Healy, K.E. (2020). Metabolically-Driven Maturation of hiPSC-Cell Derived Cardiac Chip. bioRxiv, 485169.

Kim, S.-D., Kang, G., Kim, J.-M., Ryu, J.-H., Kim, K.-C., Kwon, H.S., Kim, K.-H., Lee, H.-K., and Song, S.-W. (2017). In Vitro Evaluation of Chemicals on hERG K+ Channels in Conventional Patch-clamp Electrophysiology. Journal of Pharmacological and Toxicological Methods 88, 198-199.

Kirsch, G.E., Trepakova, E.S., Brimecombe, J.C., Sidach, S.S., Erickson, H.D., Kochan, M.C., Shyjka, L.M., Lacerda, A.E., and Brown, A.M. (2004). Variability in the measurement of hERG potassium channel inhibition: effects of temperature and stimulus pattern. Journal of pharmacological and toxicological methods 50, 93-101.

Kramer, J., Obejero-Paz, C.A., Myatt, G., Kuryshev, Y.A., Bruening-Wright, A., Verducci, J.S., and Brown, A.M. (2013). MICE models: superior to the HERG model in predicting Torsade de Pointes. Scientific reports 3, 2100.

Liang, P., Lan, F., Lee, A.S., Gong, T., Sanchez-Freire, V., Wang, Y., Diecke, S., Sallam, K., Knowles, J.W., and Wang, P.J. (2013). Drug screening using a library of human induced pluripotent stem cell–derived cardiomyocytes reveals disease-specific patterns of cardiotoxicity. Circulation 127, 1677-1691.

Martin, R.L., Mcdermott, J.S., Salmen, H.J., Palmatier, J., Cox, B.F., and Gintant, G.A. (2004). The utility of hERG and repolarization assays in evaluating delayed cardiac repolarization: influence of multi-channel block. Journal of cardiovascular pharmacology 43, 369-379.

Mathur, A., Loskill, P., Shao, K., Huebsch, N., Hong, S., Marcus, S.G., Marks, N., Mandegar, M., Conklin, B.R., Lee, L.P., and Healy, K.E. (2015). Human iPSC-based cardiac microphysiological system for drug screening applications. Sci Rep 5, 8883.

Mohammad, S., Zhou, Z., Gong, Q., and January, C.T. (1997). Blockage of the HERG human cardiac K+ channel by the gastrointestinal prokinetic agent cisapride. American Journal of Physiology-Heart and Circulatory Physiology 273, H2534-H2538.

Naito, H., Melnychenko, I., Didié, M., Schneiderbanger, K., Schubert, P., Rosenkranz, S., Eschenhagen, T., and Zimmermann, W.H. (2006). Optimizing engineered heart tissue for therapeutic applications as surrogate heart muscle. Circulation 114, I72-78.

Potet, F., Bouyssou, T., Escande, D., and Baró, I. (2001). Gastrointestinal prokinetic drugs have different affinity for the human cardiac human ether-a-gogo K+ channel. Journal of Pharmacology and Experimental Therapeutics 299, 1007-1012.

Rampe, D., Roy, M.-L., Dennis, A., and Brown, A.M. (1997). A mechanism for the proarrhythmic effects of cisapride (Propulsid): high affinity blockade of the human cardiac potassium channel HERG. FEBS letters 417, 28-32.

Redfern, W.S., Carlsson, L., Davis, A.S., Lynch, W.G., Mackenzie, I., Palethorpe, S., Siegl, P.K.S., Strang, I., Sullivan, A.T., and Wallis, R. (2003). Relationships between preclinical cardiac electrophysiology, clinical QT interval prolongation and torsade de pointes for a broad range of drugs: evidence for a provisional safety margin in drug development. Cardiovascular research 58, 32-45.

Sirenko, O., Crittenden, C., Callamaras, N., Hesley, J., Chen, Y.-W., Funes, C., Rusyn, I., Anson, B., and Cromwell, E.F. (2013). Multiparameter in vitro assessment of compound effects on cardiomyocyte physiology using iPSC cells. Journal of biomolecular screening 18, 39-53.

Sirenko, O., Hancock, M.K., Crittenden, C., Hammer, M., Keating, S., Carlson, C.B., and Chandy, G. (2017). Phenotypic assays for characterizing compound effects on induced pluripotent stem cell-derived cardiac spheroids. Assay and drug development technologies 15, 280-296.

Walker, B.D., Singleton, C.B., Bursill, J.A., Wyse, K.R., Valenzuela, S.M., Qiu, M.R., Breit, S.N., and Campbell, T.J. (1999). Inhibition of the human ether-a-go-go-related gene (HERG) potassium channel by cisapride: affinity for open and inactivated states. British journal of pharmacology 128, 444-450.

Wang, J., Della Penna, K., Wang, H., Karczewski, J., Connolly, T.M., Koblan, K.S., Bennett, P.B., and Salata, J.J. (2003). Functional and Pharmacological Properties of the Canine ERG Potassium Channel. American Journal of Physiology-Heart and Circulatory Physiology, H256-H267.

Wible, B.A., Hawryluk, P., Ficker, E., Kuryshev, Y.A., Kirsch, G., and Brown, A.M. (2005). HERG-Lite®: A novel comprehensive high-throughput screen for drug-induced hERG risk. Journal of pharmacological and toxicological methods 52, 136-145.
